# Conceptual Evolution of Cell Signaling

**DOI:** 10.3390/ijms20133292

**Published:** 2019-07-04

**Authors:** Arathi Nair, Prashant Chauhan, Bhaskar Saha, Katharina F. Kubatzky

**Affiliations:** 1 National Center for Cell Science (NCCS), Ganeshkhind, Pune 411007, India; 2 Zentrum für Infektiologie, Medizinische Mikrobiologie und Hygiene, Universitätsklinikum Heidelberg, Im Neuenheimer Feld 324, 69120 Heidelberg, Germany

**Keywords:** cell signaling, signal transduction, crosstalk, receptor, ligand, evolution

## Abstract

During the last 100 years, cell signaling has evolved into a common mechanism for most physiological processes across systems. Although the majority of cell signaling principles were initially derived from hormonal studies, its exponential growth has been supported by interdisciplinary inputs, e.g., from physics, chemistry, mathematics, statistics, and computational fields. As a result, cell signaling has grown out of scope for any general review. Here, we review how the messages are transferred from the first messenger (the ligand) to the receptor, and then decoded with the help of cascades of second messengers (kinases, phosphatases, GTPases, ions, and small molecules such as cAMP, cGMP, diacylglycerol, etc.). The message is thus relayed from the membrane to the nucleus where gene expression ns, subsequent translations, and protein targeting to the cell membrane and other organelles are triggered. Although there are limited numbers of intracellular messengers, the specificity of the response profiles to the ligands is generated by the involvement of a combination of selected intracellular signaling intermediates. Other crucial parameters in cell signaling are its directionality and distribution of signaling strengths in different pathways that may crosstalk to adjust the amplitude and quality of the final effector output. Finally, we have reflected upon its possible developments during the coming years.

## 1. Introduction

Cells receive and respond to extracellular cues through receptors. The first response is triggering complex signaling networks that relay extracellular cues into the cell, culminating in the reprogramming of various biochemical, genetic, and structural processes. The cellular signaling starts as soon as the first messenger (the ligand) binds to its receptor—a protein with the complementary structure on a transmembrane protein or within the cell. The binding of the ligand induces conformational changes to the receptor and activates well-controlled sets of reactions carried out by the second messengers or signaling intermediates that transduce the message from the receptor to the quantifiable effector functions. Thus, cell signaling is a crucial cog in the cellular response system. The discovery of cellular signaling dates back to 1855 when Claude Bernard described how certain ‘internal secretions’ of ductless glands, released into the bloodstream, can have effects on distant cells. Around 1880, British naturalist Charles Darwin and his son Francis Darwin discovered a similar phenomenon of phototropism of coleoptile (shoot tips) in plants and inferred “Some influence is transmitted from the tip to the more basal regions of the shoot thereby regulating growth and inducing curvature” [1]. This transmittable factor or messenger was later termed as auxin. A few years later, John Langley and his student Thomas Elliott discovered a ‘receptive substance’ or receptors while studying sympathetic neuro-effector transmission [2]. Later in 1905, Ernest Starling first coined the word ‘hormone’ (*Gr.*, arousing or excite) to explain, “The chemical messengers which spread from cell to cell along the bloodstream, may coordinate the activities and growth of different parts of the body” [3]. Following the discoveries of the messengers and receptors, the downstream intracellular events started unfolding during the 1950s. Rita Levi-Montalcini discovered that tumor extracts can cause neurite outgrowth and identified the factor as the nerve growth factor (NGF) [4]. The discovery of inositol phosphate pathway [5], phosphorylation-dependent proteins [6], and the finding that skeletal contraction occurs on injecting Ca^2+^ into the cells and that binding of adrenaline and glucagon to cellular receptors leads to the generation of cyclic adenosine monophosphate (cAMP) [7], further unraveled the details of cellular signaling. This was followed by the discovery of epidermal growth [8], G-proteins [9], tumor necrosis factor [10], and of a retroviral Src protein that functions as a tyrosine-specific kinase [11]. All these discoveries led to an enhanced understanding of how cells receive, perceive, and decode the signal.

The word ‘signal transduction’ appeared in biological literature in the 1970s [12], further elucidation of which was provided by Martin Rodbell in 1980 who postulated that ‘individual cells were cybernetic systems made up of three distinct molecular components: discriminators, transducers and amplifiers.’ The cell receptors are the discriminators that receive external signals and process this information across the cell membrane via the cellular transducers. The intensification of the signal happens through amplifiers that relay signals within or across the cells. Signal transduction is not a linear sequential activation cascade of signaling molecules, but rather a nexus of signaling relays within the cell. Cells perceive extracellular signals, which are processed and interpreted by the intracellular machinery in a well-defined manner. In some cases, the conformational change in the ligand-bound receptor activates its kinase activity triggering the downstream signaling. Whereas in other cases, the ligand-bound receptors recruit adaptors that engages a number of signaling intermediates, primarily kinases, to form a signalosome (CSN) complex. This relays the signal further through various other second messengers such as calcium (Ca^2+^), cAMP, cyclic guanosine monophosphate (cGMP), diacylglycerol (DAG), inositol triphosphate (IP_3_), kinases, lipids derivatives, phosphatases, etc. Such ‘second messengers’ crosstalk amongst them, integrate diverse information, and relay it to the target molecules in the cytosol and/or nucleus triggering the effector functions.

## 2. Components of Cell Signaling

### 2.1. Ligands or Signals

**Figure 1:**
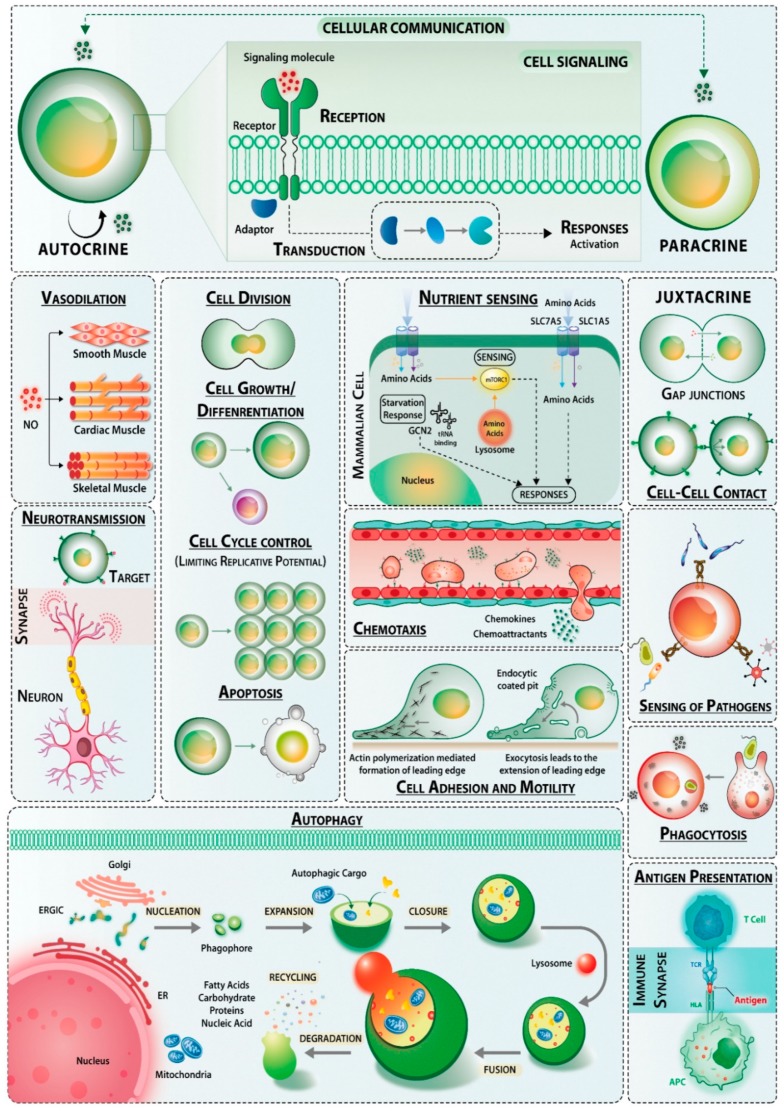
Cellular signaling controls various aspects of multicellular life forms. Not only the key biological processes such as cell division, differentiation, growth, and cell-cycle transition, but also specialized cell-specific functions such as neurotransmission, pathogen-sensing, phagocytosis, and antigen-presentation are controlled by specific signaling pathways. The process of autophagy and nutrient cycling and recycling are some accessory pathways that are triggered by definitive signaling cues.

Signals are perturbations of cellular homeostasis and cells mainly respond to mechanical (mechanotransduction), electrical (electrotransduction), or chemical (chemotransduction) stimuli. In biology, the majority of the signals are chemical in nature. For example, prokaryotic cells have sensors that detect nutrients and mediate mechanotransduction towards higher nutrient gradients. Similarly, eukaryotic cells also have sophisticated ways of responding to signals such as growth factors, hormones, cytokines, neurotransmitters, extracellular matrix components, etc. There are different types of signaling, which may be characterized as endocrine (long-range communication), paracrine (short-range/localized), juxtacrine (contact-dependent signaling), autocrine (acting on the same cell that produces the factor), and neuronal-neurotransmitter mediated (signaling at synaptic junctions). The chemical nature of the ligands is diverse including small molecules such as lipids (e.g., prostaglandins, steroids), proteins (e.g., peptide hormones, cytokines, and chemokines, growth factors), complex polymers of sugars (e.g., β-glucan and zymosan), and their combinations (e.g., proteoglycans), nucleic acids, etc. Peptide ligands are polar in nature and bind to cell-surface receptors while steroids being lipophilic in nature diffuse passively across the cell membrane. Once inside the hydrophilic cytosol, carrier proteins assist trafficking of steroids to the nuclear receptors. Similarly, nitric oxide, owing to its small size, diffuses across the plasma membrane and activates pathways regulating vasodilation. An overview of the different modes of cellular responses driven by a multitude of signaling cascades is depicted in Figure 1.

### 2.2. Receptors

**Figure 2:**
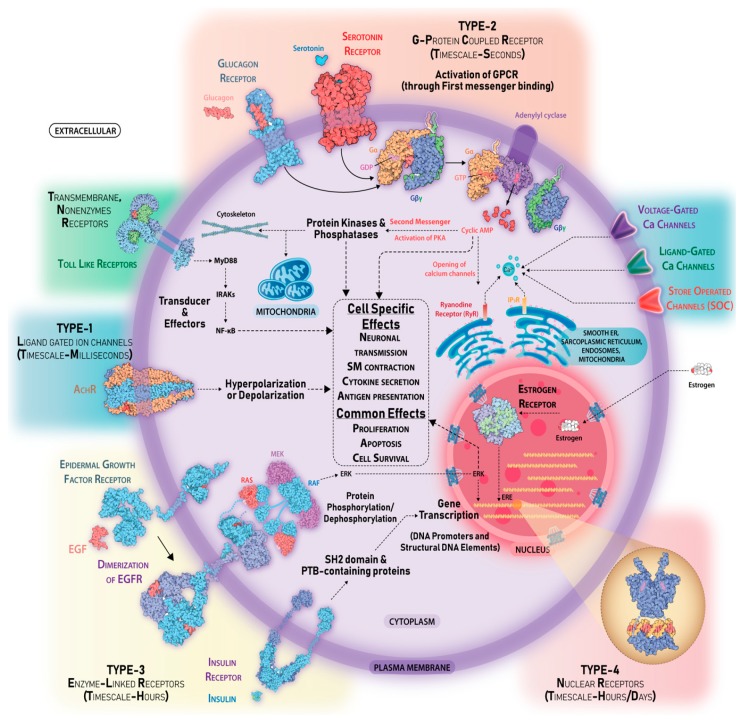
A holistic view of the various cell-surface and intracellular receptors, their associated intracellular components, and downstream effects (Note—Receptors and protein structures are adapted from PDB-http://pdb101.rcsb.org).

There are two broad categories of receptors cell-surface receptors and intracellular receptors. Cell-surface receptors span the plasma membrane and have distinct extracellular ligand binding domain, a transmembrane domain that is hydrophobic in nature, and a cytoplasmic domain. Upon ligand binding, the membrane-spanning receptors undergo a conformational change in their extracellular domain and activate the cytoplasmic domain-linked enzymatic machinery, usually kinases, phosphatases, and adaptors. These may be covalently linked to the receptor and may produce second messengers for subsequent transduction of the signal. Cell-surface receptors can be categorized into G-protein coupled receptors, ionotropic receptors, and receptor tyrosine kinases (Figure 2, receptor subclasses are omitted for simplicity).

Intracellular receptors may be nuclear receptors (for e.g., androgen receptor, estrogen receptor, glucocorticoid receptor, progesterone receptor, retinoic acid receptor thyroid receptor, etc.), cytoplasmic receptors, or organellar receptors like in mitochondria, endoplasmic reticulum (ER), and Golgi apparatus (subcellular compartments) that bind to small lipophilic molecules, which cross the plasma membrane. For example, receptors for glutamate [13], thyroid hormone, estrogen, and androgens are also present on the mitochondrial membrane [14]. Sigma receptors are found to be associated with the ER membrane and act as a chaperone to stabilize ER membrane proteins like IP_3_ receptor [15]. Brown et al. found the presence of the mannose-6-phosphate receptor in *cis* Golgi cisternae that binds to lysosomal enzymes bearing Man-6-P recognition marker [16].

### 2.3. Specificity in Signaling

Receptors exhibit a high binding affinity for their specific ligands, e.g., the insulin receptor has a high binding affinity for only insulin, conferring specificity to signaling. Interestingly, varying cell types might have a different number and type of receptors, whereby some cell types might be devoid of some specific receptors while others may be enriched in a particular type of receptor. In some cases, receptors responsible for signal detection may form clusters on apical/basal surfaces of the cell to produce a heightened response as observed in epidermal growth factor receptor (EGFR) signaling [17].

Formation of the immune synapse (IS) presents a very interesting example of co-clustering of the T cell receptor (TCR) and adhesion and costimulatory receptors within a confined spatial region on the plasma membrane. Signaling at IS is initiated as soon as ligation of an antigen-presenting cell (APC) occurs by its physical contact with lymphocytes (via cognate receptor–coreceptor pairs). Briefly, endocytic signaling mediates protein targeting to the naïve T cells IS. T cells become transiently polarized as a result of the translocation of microtubule organizing center (MTOC or centriole) beneath the contact region of the T cell and the antigen-presenting cell (APC) [18]. The regulation of signal transduction occurs via the lateral compartmentalization of membrane proteins into distinct microdomains. TCR signaling initiates recruitment of the mediators Lck (lymphocyte-specific protein tyrosine kinase) and LAT (linker for activation of T cells). However, a microdomain-localized cluster of differentiation (CD) 45 inactivates lymphocyte-specific protein tyrosine kinase (Lck) and inhibits TCR signaling at the early IS. The counterbalancing activity of galectin lattice and actin cytoskeleton negatively and positively regulates Lck activity in resting T cells. In addition to this, such counterbalancing activities also affect CD45 versus TCR clustering and signaling at the early IS [19]. Lck assembly at the TCR cluster site and its entry and exit from the cluster domain can be monitored by fluorescence microscopy [20]. Using photoactivated-localization microscopy (PALM) imaging of individual LAT molecules, Sherman et al. showed that LAT and TCRζ exist in overlapping regions. Within such regions, nanoscale domains exists that could function as the prime spots for T cell activation [21]. Receptor clustering is not only limited to immunological receptors such as B cell receptor (BCR) [22] or the FcεR1 [23], but also extends to other cells and receptors such as EGFR [17].

#### 2.3.1. Lipids in Signaling

Another tier to signaling specificity is added by lipid microdomains that can selectively recruit and exclude signaling components. The specificity of signaling is enhanced due to receptor localization into microdomains that have specific sets of signaling constituents. Hence, lipid microdomains serve as organizing centers for signaling molecules and prevent signal interference and non-specific signaling. All the necessary protein complexes are co-localized spatially in close proximity to each other and, thus, signal interference can be minimized. Discrete microdomains that span over nanometer scale (10–200 nm) within the plasma membrane (PM) are known as ‘lipid rafts’. Such lateral fragments in PM are rich in cholesterol, glycophospholipids, and glycosylphosphatidylinositol (GPI)-anchored proteins [24]. This intricate organizational heterogeneity in PM fosters protein–protein, protein–lipid, and lipid–lipid interactions. Although microdomains are characterized by an abundance of cholesterol, cholesterol-independent rafts also exist [25]. Receptor clustering, distribution, and density are some key spatial features of cellular signaling that occur within these rafts, and influences parameters like propagation, strength, and effectiveness of signals [26]. Evidently, many receptor systems employ receptor clustering for initiating transmembrane signaling. For example, Grassmé et al. showed that acid sphingomyelinase (ASM) is crucial for the clustering of CD40. Using fluorescent microscopy, they showed that extracellularly oriented ceramide is released by the action of ASM, which mediates clustering of CD40 in membrane domains rich in sphingolipids [27].

Lipid rafts may also serve as redox signaling platforms. For example, the Nox (NADPH oxidase) multi-subunit enzyme complex is a well-known mediator of redox signaling in leukocytes and endothelial cells (ECs). Formation of the Nox signalosome in ECs allows them to drive redox signaling, which is important in redox regulation in cells and organs by the production of O_2_^−^. Cholesterol-depleting molecules such as methyl-β-cyclodextrin (M-*β*-CD) or filipin abrogate membrane raft clustering and abolish the Nox-subunit assembly [28]. The EGFR–Ras–Raf (rat sarcoma-rapidly accelerated fibrosarcoma) pathway also exhibits a reversible association with raft signaling microdomains. Rafts mediate important biological functions including signalosome assembly, caveolae- or clathrin-dependent endocytosis, and sorting of polarized cells by affecting protein motif-based differential partitioning. It also plays important roles in immune cells like facilitating the immune receptor (TCR/BCR) signaling and may contribute towards viral dissemination and budding during infections [29].

In addition to the signalosome assembly at the PM, certain intracellular signalosomes also exist. The COP9 (constitutive photomorphogenesis 9) signalosome is another well-studied signalosome known to control the ubiquitin–proteasome system through regulation of Cullin-RING-E3 ubiquitin ligase activity by deneddylation. The COP9 signalosome inhibits Cullin-RING-E3 ubiquitin ligase by removing Nedd8 (neural precursor cell-expressed developmentally downregulated-8) modifications from their Cullin subunits [30]. The COP9 signalosome is also reported to regulate selective autophagy by regulating the expression of Rab7, and Csn8/CSN that play a critical role in autophagosome maturation [31].

Lipids and their derivatives are versatile signaling molecules, for example, inositol lipids and inositol phosphates play an important role in cell signaling. A variety of phosphoinositol phospholipids are generated by the action of lipid kinases and phosphatases on the inositol ring of inositol phospholipids. Lipid kinases can be activated by a variety of stimuli. For example, when mammalian neurotrophins family members like nerve growth factor (NGF) bind to tyrosine kinase receptors (Trk), tyrosine residues in its cytoplasmic domain get autophosphorylated. This binding causes the activation of the Ras/mitogen-activated protein kinase (MAPK) pathway, which leads to increased proliferation and cell growth through the extracellular signal-regulated kinase (ERK) pathway. The activated TrkA receptor binds to and activates phospholipase C-γ (PLC-γ), which cleaves membrane-bound phosphatidylinositol-bisphosphate (PIP_2_) to yield IP_3_ and DAG. IP_3_ translocates from the plasma membrane to its receptors InsP_3_R that is coupled to a Ca^2+^-release channel in the ER membrane, where it stimulates the release of Ca^2+^ from ER stores resulting in mobilization of Ca^2+^. Cytosolic Ca^2+^ is thus accessible to calmodulin and other Ca^2+^-binding cytoskeletal proteins. These proteins may be involved in the formation of microtubules and intermediate filament. Many of the enzymatic effects of the released Ca^2+^ are mediated through protein phosphorylation catalyzed by a family of Ca^2+^/calmodulin dependent protein kinases (CaMK-II/IV). The binding of Neurotrophin-3 (NT-3) to TrkC causes sequential activation of the PI3K/Akt pathway, thus preventing apoptosis and increasing cell-survival. On the other hand, TrkB transduces the brain-derived neurotrophic factor (BDNF) signal through Ras-ERK, PI3K, and the PLC-γ pathway resulting in cell survival and differentiation [32].

Another diverse lipid-derived signaling molecule is the phosphatidylinositol-3-kinases (PI3Ks) that generate phosphatidylinositol-3,4,5-trisphosphate (PIP_3_) by phosphorylating phosphatidylinositol-4,5-bisphosphate (PIP_2_) at the 3′-position of the inositol ring. In mammals, four class-I PI3K isoforms have been reported that play non-overlapping roles. The class-IA PI3Ks are heterodimers composed of a catalytic subunit (p110α, p110β, p110δ) and regulatory subunits (p85); the regulatory subunit mediates receptor binding, activation, and localization. PI3Ks are activated upon growth factor (PDGF) stimulation via receptor tyrosine kinases [33]. Isoform p110δ expression is restricted to immune cells whereas p110α and p110β are universally expressed [34]. Given the importance of class-I PI3Ks in the maintenance of cellular homeostasis, mutations in class-I PI3Ks or in p85 adaptors lead to several diseases ranging from metabolic syndromes to cancer. Two diverse signaling pathways of actin polymerization and chemoattractant sensitivity are observed in *Dictyostelium discoideum* through two independent effectors PI3K and phosphatase and tensin homolog (PTEN) that act reciprocally. PI3K can initiate pseudopod formation [35] while PTEN plays a suppressive role in lateral pseudopod formation [36], which exemplifies the reciprocal action of kinases and phosphatases in cellular mechanobiology.

#### 2.3.2. Signaling Domains

Signaling specificity of a protein often arises from its domain structure. A domain is a distinctly folded part of a protein that imparts particular functions and allows it to differentially engage in signaling pathways. Proteins that are functionally alike can have distinct domains and vice versa. Domains can be of variable length ranging from 50–300 amino acid residues (aa) [37]. Domains organize the functional units of a protein, for example, the EF-hand domain in protein Calmodulin. In addition, domains can be swapped by genetic manipulation between proteins to generate chimeras or mutants. Domain shuffling by recombination events gives rise to proteins with new domain arrangements, further diversifying protein functions [38]. The Pfam database annotates and houses domains of known and unknown biological functions [39]. Janin and Wodak proposed that structural coordinates of domains within a protein contain more interactions within itself than with the rest of the protein [40]. Protein domains also define the stabilizing conformations for optimal protein folding.

#### 2.3.3. Common Signaling Domains Found in Proteins

**Figure 3:**
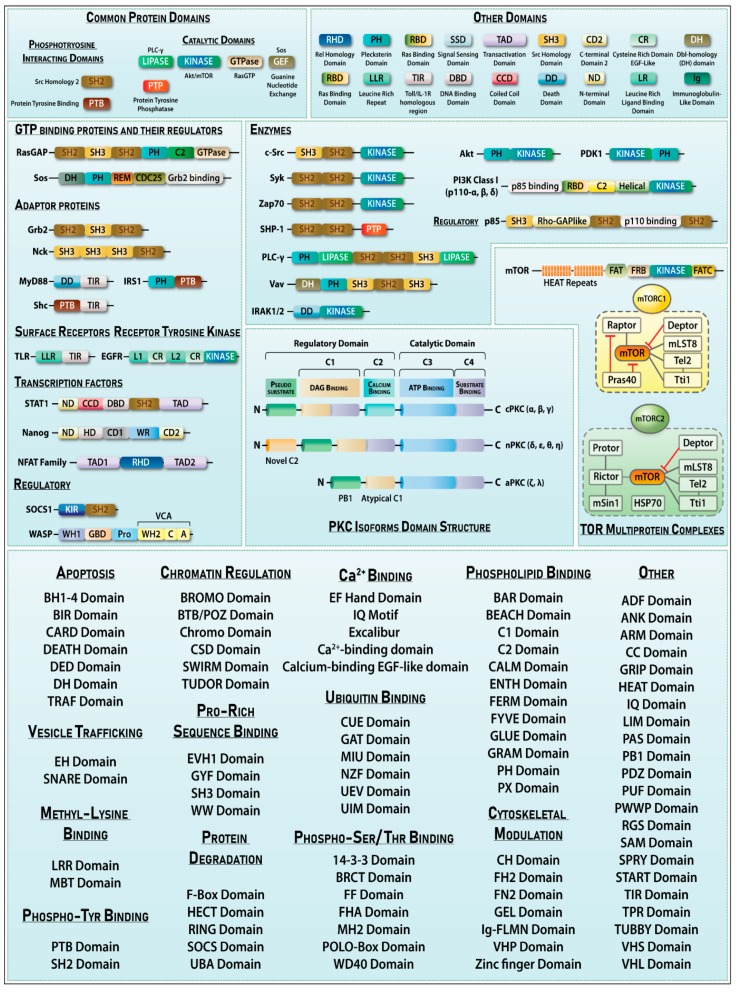
Represents the different protein domains and their categorization based on binding with other molecules and their biological functions. (Note—Sources: http://www.ebi.ac.uk/interpro/; http://www.cellsignal.com).

Src homology domain (SH3) consists of short (~50aa) residues that allow protein–protein interactions to occur. SH3 domain comprises of five to eight β-strands arranged into two antiparallel β-sheets or in a β-barrel, which plays an important role in recognizing cognate binding partners. SH3 domains are commonly present in proteins regulating cytoskeletal changes, PI3K, Ras GTPase-activating proteins, CDC24, myosin, and phospholipase C, etc. Another signaling domain, SH2, represents a phosphotyrosine selective recognition domain [41]. The SH2 domain docks tightly to a subset of phosphorylated tyrosine residues containing proteins, which are generated by the catalytic action of kinases [42]. The Pleckstrin homology domain (PH) is another domain commonly found in proteins that are intracellular signaling components or function as cytoskeletal components. This domain has an affinity for phosphatidylinositol lipids (PIP_3_ and PIP_2_) localized within membranes and some G-proteins [43]. PIP_2_ is required for the function of both phospholipase D [44] and ARF [45]. Pleckstrin domain-containing proteins also include Ser/Thr kinases like the Akt/Rac family. Caspase-8 and Caspase-9 interact with adaptors via death effector domain (DED) and trigger an auto-activation caspase cascade [46]. Basic Leucine zipper domain (bZIP domain) is the most commonly occurring protein in eukaryotic DNA-binding proteins. Immunoglobulin-like domains are commonly occurring domains in immunoglobulin superfamily proteins and are important in processes of cellular adhesion, activation, and molecular interaction [47]. Figure 3 shows the various signaling domains found in signaling-associated proteins.

### 2.4. Signal Transducers

Upon ligand binding, the receptor undergoes a conformational change and its cytoplasmic transducers or adaptor proteins are activated depending on the type of receptor. However, the mechanistic operation of each type of receptor varies. GPCR, on ligand binding, undergo a conformational change wherein its associated heteromeric G proteins are activated by guanosine triphosphate (GTP) binding [48] (Figure 2). Once active, it dissociates into G_α_ and G_βγ_ subunits and G_α_ can activates a range of second messengers such as cAMP [49], Phospholipase C (PLC) [50], Rho GEFs [51], etc. G_βγ_ can activate G-protein-regulated inwardly rectifying potassium (K^+^) channels (GIRKs) [52], P/Q- and N-type voltage-gated Ca^2+^ channels [53], PI3K isoforms [34], PLC isoforms [54], and adenylyl cyclase isoforms [49]. GPCRs may also function independently of G-proteins via the G protein-coupled receptor kinase (GRKs), β-arrestin [55], and Srcs [56]. In addition to heteromeric G-proteins, monomeric small GTPases, namely Ras [57], the Ras-homologous (Rho) protein [58], Ras-associated binding (Rab) proteins [59], ADP ribosylation factor (Arf) [60], and Ras-related nuclear protein (Ran) [61], serve as important signal transducers in different signaling pathways. Guanine nucleotide exchange factors (GEFs) activate the GTPase through guanosine diphosphate (GDP)/GTP replacement. Upon activation, the GTPases interact with its specific downstream effectors.

Enzyme-linked receptors like receptor tyrosine kinases undergo dimerization upon binding to their ligand, which leads to the activation of the cytoplasmic domain by transphosphorylation of tyrosine residues. This phosphorylation creates binding sites for Src homology 2 (SH2) domain and phosphotyrosine binding (PTB) domain-containing proteins, which concomitantly activates Src and PLCγ [62]. Amino acids like serine, threonine, and tyrosine are most susceptible to a phosphorylation-dependent modification. Addition of a phosphate group to these residues causes a conformational change in the enzyme, further activating or inhibiting it. Protein phosphatases however, work reciprocally to the kinases by removing the phosphate group from the enzymes thereby reversing the effect. Phosphorylation is, thus, nature’s way of fine-tuning the function of proteins. A single second messenger or signaling intermediate can play a role in multiple signaling pathways. This leads to ‘redundancy’ and ‘parallelism’ in the process of integration of information from external inputs. Not only receptor tyrosine kinases, but also receptors that lack an intrinsic kinase activity undergo clustering and conformational changes. For instance, on binding to membrane-bound or polyvalent soluble antigen, BCR undergoes rapid oligomerization [63] followed by conformational changes in the cytoplasmic domain of BCR from a closed to an open form, hence permitting its association with kinases [64].

In the case of ionotropic receptors (ligand-gated ion channels), upon binding of the ligand to the extracellular domain, a conformational change occurs that open the transmembrane ion pore, a channel through which Na^+^, K^+^, Ca^2+^, and/or Cl^−^ ions are conducted at a rate of up to ~10^7^ ions/second into the cell. Ion-binding enzymes and voltage-sensitive channels respond to this influx of ions and generate a response. The canonical states occupied by most ligand-gated ion channels are resting, activated, and desensitized states. Therefore, their manipulation by the chemical stimulus is possible. For example, in the case of glutamate type-2 (GluR2) receptor, upon desensitization, decoupling of agonists from the ion channel gating occurs, which leads to conformational rearrangements [65]. Another example is the rapid synaptic transmission of AMPA-type glutamate receptors.

In Hippocampal neurons, transmembrane α-amino-3-hydroxy-5-methyl-4-isoxazolepropionic acid (AMPA) receptor regulatory proteins present a variable stoichiometry depending on the cell type. AMPARs in hippocampal pyramidal cells contain more transmembrane AMPA receptor regulatory proteins (TARPs) than those in dentate gyrus granule cells, therefore, the regulatory mechanism for AMPA activation may be regulated intrinsically in a cell-type-specific manner varyingly by different regulatory proteins [66]. Interestingly, not only these receptors can be manipulated by electrical or chemical impulses but also optogenetics, along with photoswitchable-tethered ligands (PTLs), allow one to closely monitor the reversible and reproducible activation or blockade of specific neurotransmitter-gated receptors and ion channels in specific cells. A spatial and temporal manipulation approach of GluRs using photorelease-caged compounds can be used to regulate chemistry and cellular physiology [67]. Optogenetics-controlled neuronal firing shows wide-range control of neuronal activity that is artifact-free and can be continued over a wide timescale [68]. Another type of voltage-gated channels is the T-type low-voltage-gated calcium channels (VGCC) that are activated through the influx of sodium resulting in Ca^2+^ influx, depolarization, and increased neuronal firing. However, the ligand-gated influx of Cl^−^ through γ-Aminobutyric acid type A (GABA_a_) receptors result in hyperpolarization and neuronal inhibition [69]. Patch-clamp studies in channel proteins revealed specific residues within ion-channel proteins that modulate the biophysical properties of the channel [70]. These insights also reveal that therapeutic targeting of such receptors is possible by understanding their roles in abnormal physiologies, whereby techniques like imaging, electrophysiological recordings, and genetic manipulation play coalescing roles in uncovering their mechanistic action.

### 2.5. Second Messengers

Once the signal is relayed to the transducers, it activates specific effectors that generate small molecules called second messengers. Common second messengers are cAMP, cGMP, IP_3_, diacylglycerol, calcium, etc. The enzyme adenylyl cyclase, when activated rapidly, converts ATP to cAMP that accumulates in the cytosol. Thus, cytosolic cAMP concentrations rise rapidly if left unrestrained and under such conditions, all the effectors of cAMP have an equal chance of being activated, which can be detrimental to the normal functioning of the cell. For this reason, cAMP levels in the cells are regulated by a class of ubiquitously expressed phosphodiesterases (PDEs) [71]. Ions are indispensable in cell signaling and any dysregulation in their concentrations can affect a number of signaling processes. For example, alterations in the homeostasis of Ca^2+^ ions is associated with neurological disorders [72] and other diseases. However, the cell stringently tries to maintain homeostatic ionic fluxes within the cytoplasm by employing active efflux pumps that pump ions back and forth from the plasma membrane and intracellular organelles. The ions traverse back to form a gradient, initiating a signaling cascade.

Ca^2+^ is a versatile ‘second messenger’ that plays a crucial role in cellular physiology. Ca^2+^ signaling has been extensively studied in the context of development and embryogenesis. Some primitive observations date back to the 1950s, when it was observed that phorbol ester and sperm activate mouse oocytes by inducing sustained oscillations in cell’s Ca^2+^ level [73]. In the following years, many remarkable discoveries were made wherein the indispensable role of Ca^2+^ signaling during fertilization and early embryogenesis was described. For example, Karl Swann et al. showed that sperm-specific Phospholipase C (PLC)-ζ triggered intracellular Ca^2+^ oscillations were responsible for activating mammalian eggs at fertilization [74]. Calcium controls numerous functions including regulation of Protein Kinase C isoforms, Ca^2+^/Calmodulin-dependent T cell activation, phases of cell cycle, and acts as a cofactor for metabolic enzymes. Moreover, it also controls calcium-dependent nitric oxide synthases (NOS). Cells use active energy (ATP) to efflux Ca^2+^ ions to maintain a physiological intracellular gradient (<10^−7^ M with an extracellular concentration of about ~1.5 × 10^−3^ M) [75]. Organelles like mitochondria, ER, and sarcoplasmic reticulum are depots of intracellular Ca^2+^ storage. Calsequestrin, on the other hand, is a major calcium-storage protein that can bind ~50 calcium ions and keeps Ca^2+^ stored in sarcoplasmic reticulum/ER along with other proteins [76]. Calcium signaling is coupled to IP_3_ that play an important role in the release of sequestered intracellular calcium. The signaling wave that initiates when cytoplasmic Ca^2+^ is liberated from intracellular depots is commonly mediated by inositol triphosphate (InsP_3_). InsP_3_ also mediates the release of other signaling intermediates like nicotinamide adenine dinucleotide phosphate (NADP), Cyclic ADP-ribose (cADPR), and sphingosine-1-phosphate (S1P) [77]. Lipid kinases and phosphatases act on the inositol ring and generate a variety of phosphoinositol-phospholipids. The functioning of Ca^2+^ ions is primarily mediated by the ubiquitous small adaptor protein calmodulin and other Ca^2+^ -binding proteins. Calmodulin can bind up to four calcium ions at its EF-hand motif and it undergoes a conformational change whereby hydrophobic methionine residues become exposed enabling its binding to basic amphiphilic helices (BAA helices) to activate a large number of target proteins [78]. Table 1 shows some important proteins that are controlled by calcium signals in diverse cell types.

DAG is an essential activator of Akt, Ras, and NF-κB signaling [100] and contributes to T cell effector functions. Diacylglycerol kinases (DGKs) are a family of enzymes that catalyze the conversion of DAG to form phosphatidic acid (PA). T cells abundantly express DGK isoforms α and ζ. Simultaneous genetic ablation of DGKα and DGKζ genes in mice shows a severe defect in thymocytes development, which is absent in mice deficient in either DGKα or DGKζ alone. This observation suggests that DGK kinases play redundant roles in T cell biogenesis [101]. The distinct domain architecture in DGKα or DGKζ suggests that differential regulation of these molecules may be responsible for directing their isoform-specific functions and their redundant roles [102] during thymocytes development.

Conventional PKCs (cPKC) act as a molecular machine for decoding Ca^2+^ and DAG signals within the cytosol [96,103]. Since cPKC is dependent on Ca^2+^ for its activation [104], Ca^2+^ signaling leads to the activation of specific isoforms of PKC that further drive lineage-specific transcription programs. For example, PKCζ is critical for IL-4 signaling and Th2 differentiation [105]. PKC family members are involved in governing various aspects of T cell biology ranging from adhesion, effector differentiation, IL-2 secretion, proliferation, apoptosis, migration, and IgG switching in B cells. Therefore, PKC isoforms along with the vital second messenger Ca^2+^; play indispensable roles in TCR-induced T cell clonal expansion and cytokine production. These cytokines include both pro- and anti-inflammatory cytokines that are produced during pathogenesis [106]. PKC signaling modulates translation efficiency and mRNA stability of the synergistic cytokines IL-2, TNF-α, and IFN-γ in a transcript-specific manner. In effector CD8^+^, T helper, and memory T cells, the production of TNF-α occurs through PKC-induced recruitment of mRNA to polyribosomes [107]. Non-specific inhibitors of Ca^2+^ binding proteins and inhibitors of PKC block the cytocidal activity of macrophage in a dose-dependent manner. PKC activation and mobilization of intracellular Ca^2+^ are key steps in the pathway of IFN-γ-dependent induction of non-discriminatory tumoricidal activity in macrophages [108].

### 2.6. Transcription Factors

**Figure 4:**
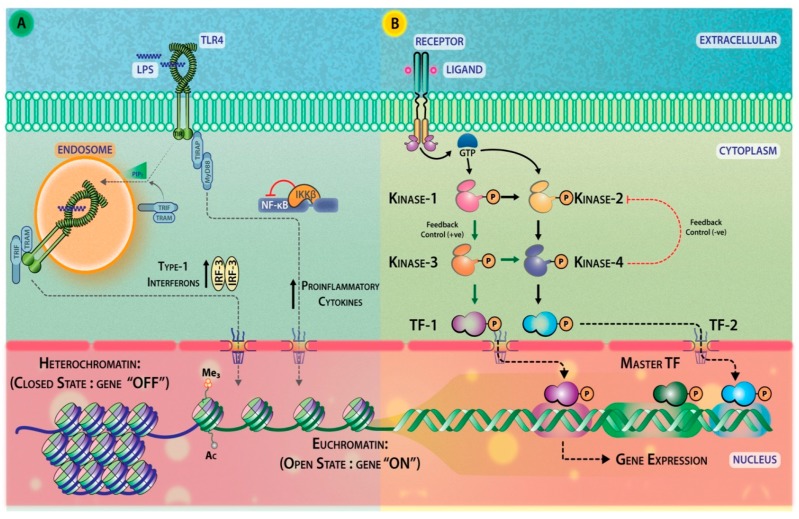
(**A**) Binding of the ligand to a receptor on the plasma membrane or its translocation to endosomes can trigger diverse signaling outputs. For example, binding of LPS to TLR4 receptor on the plasma membrane or on its translocation to the endosome can lead to the activation of distinct signaling intermediates and eventually specific transcription factors. (**B**) A variety of molecules (solid- adhesion molecules, extracellular matrix components; soluble factors) can elicit cell signaling. Receptor-ligand coupling triggers the enzymatic machinery that activates various transcription factors, which regulates gene expression.

The ultimate target of signaling relays are the transcription factors that regulate gene expression and eventually allow transforming the received signal into a change of cellular activity. Some allosteric effectors can bind to regulatory proteins (like transcription corepressors/enhancers), which may modulate the gene expression. Some members of the E2F family of transcription factors (E2F7), methyl-CpG-binding protein 2 (MeCP2), and CBF-1, suppressor of hairless, Lag-1 (CSL) are known examples of transcription factors that are regulated this way. Signaling-dependent transcription factors can be further classified into: (1) Steroid receptor family (e.g., RXR, PPARs), (2) transcription factors activated by internal signals (p53, SREBP, orphans), and (3) cell surface receptor-ligand activated transcription factors, which can be further subdivided into a) resident nuclear factor (CREBs, Fos/Jun, MEF-2) and b) latent cytoplasmic factors (STATs, SMADs, NF-κB/Rel, NOTCH, NFAT, etc.) [109]. In eukaryotes, like most other proteins, transcription factors are transcribed in the nucleus but are then translated in the cytoplasm. Therefore, many transcription factors that are active in the nucleus also contain a nuclear localization sequence that directs them to the nucleus [110]. The downstream outcome of signaling events may be diverse ranging from survival, proliferation, differentiation, stress responses, senescence, or apoptosis. Figure 4 illustrates the spatial segregation of signals that occurs through various platforms. For example, TLR4 signals through its ligand (lipopolysaccharide-LPS), from the plasma membrane where it associates with adaptors TIRAP and Myd88 and activates NF-κB [111]. Alternatively, when the LPS-bound-TLR4 signalosome translocates to the endosomal compartment, it associates with adaptor TRAM and activates Type-1 interferon signaling pathway, in particular IFN-β through transcription factor Interferon regulatory factor 3 (IRF-3) [112].

## 3. Directionality of Signaling

The directionality of signal transduction does not necessarily arise from the plasma membrane. Key cellular molecules like lipids, proteins, and other molecules are transported within the cell by endomembrane system. This process follows a well-defined route for the transportation of proteins containing cargo on polymeric cytoskeletal networks [113]. Two important proteins in this regard are Kinesin and Dynein that transport cargo across microtubule networks within cells [114]. In addition to signaling that originates from the plasma membrane, compartmentalized signaling from subcellular locations allow the signaling to achieve further ramification. This compartmentalization process allows the cells to spatially confine and segregate molecules of particular signaling pathway within the cells that require a specialized niche for signaling. For example, the lysosomal compartment requires a lower pH as compared to the cytosol to facilitate the degradation of targeted material and its presentation for immune activation via MHC-II. Their analogous structures like melanosomes and phagosomes also have very specialized endogenous signaling environments. Each subcellular organelle like ER, Golgi, or mitochondria may have an autonomous behavior and may inter-regulate each other’s functions. For example, mitochondrial fission, distribution, and autophagy are regulated by their organellar contact with the ER [115]. Similarly, the contact between mitochondria and lysosome allow bidirectional regulation of mitochondrial and lysosomal dynamics [116]. In this regard, the regulated supply of proteins or other signaling components to the compartment becomes important because otherwise the overall functioning of the system will be restricted. With numerous proteins and even more signaling intermediates participating in simultaneous subcellular signaling pathways, the formation of compartments for effective transport of proteins and molecular components either by passive diffusion or by direct recruitment becomes necessary. The directionality of signaling is afforded by the spatiotemporal distribution of the signaling molecules within the cell and by scaffolding proteins that pre-assemble the signaling components and prepare them for recruitment to the site of activation, thus eliminating physiologically irrelevant signals. For example, the compartmentalization of protein kinase A (PKA) signaling at mitochondria by large multi-domain scaffold proteins A-kinase anchor proteins (AKAPs) occurs at dendrites, dendritic spines, cytosol, and axons in neurons [117]. cAMP molecules activate PKA and AKAP that acts as a scaffolding protein to assemble relevant molecular components [118].

### 3.1. Retrograde and Anterograde Signaling

Most of the proteins required for the functioning of mitochondria and plastids are encoded in the nucleus with the exception of a few that are encoded by the genome of the organelle (organellar gene expression, OGE). A coordinated mechanism of regulation exists between the OGE and the nuclear gene expression NGE. Retrograde signaling occurs when organelles (mitochondria and chloroplast) relay specific information to the nucleus that modulates the expression of nuclear genes. Contrary to this, anterograde signaling refers to the coordination by nuclear-encoded factors over organellar gene expression. Retrograde signaling in chloroplast can be related to biogenic control (photosystem) or operational control (adaptation and response to changes in the environment) [119]. In addition to the redox state of the organelle, the tetrapyrrole biosynthesis pathway, ROS, and organellar gene expression (OGE), chloroplast metabolites also are known to regulate nuclear gene expression (NGE) [120,121]. Moreover, transcription factors released from the chloroplast to the nucleus also modulate NGE [122].

Retrograde signaling from mitochondria to the nucleus is of immense importance as it exemplifies intra-organellar communication [123]. Activation of an estimated 400 nuclear genes can be triggered by dysfunctional mitochondria [124]. A peroxisomal citrate synthase *Cit2* is one of these genes and is often considered as a marker for altered mitochondrial function [125]. Signals elicited by dysfunctional mitochondria induce stress responses and lead to impaired ATP production. Thus, low cytosolic ATP concentration favors retrograde response (Rtg)–Mks1 interaction [126] that allows for Rtg1–Rtg3 activation (RTG-dependent signaling). Rtg1 acts both as a positive and a negative regulator of retrograde response and Rtg2 as a transducer of mitochondrial signals that affects the phosphorylation state and subcellular localization of Rtg3 [127]. In mammals, mitochondrial dysfunction translates into a drop in the membrane potential (mΔΨ). This causes increased Ca^2+^ concentrations in the cytosol. Consequently, calcium-dependent kinases and phosphatases are activated leading to the activation of different transcription factors. In yeast, retrograde signaling pathways associated with mitochondria are extensively mined. Other signaling pathways activated from mitochondria are associated with cell death through the release of cytochrome-C and caspase-dependent cell death and by the release of reactive oxygen species (ROS). Mitochondria outer membranes serves as a scaffold for signaling complexes [128].

The nuclear genes code for most proteins of the respiratory apparatus and enzymes required for biochemical functions as the mitochondrial DNA has a limited coding capacity. Mitochondrial functions closely rely on bidirectional mitochondrial–nuclear communication and retrograde signaling through the mitochondria to the nucleus might add a new tier of regulation of mitochondrial gene transcription. G-protein pathway suppressor (GPS) 2 is a mediator of mitochondrial retrograde signaling. It also acts as a transcription activator of mitochondrial genes encoded by the nucleus. GPS2 is crucial for mitochondrial biogenesis [129]. Apart from retrograde and anterograde signaling from the mitochondria to the nucleus and from the nucleus to mitochondria, respectively, many other types of intraorganellar communication are known. A befitting example of intraorganellar communication is by learning about a protein complex known as ERMES (endoplasmic reticulum (ER)-mitochondria encounter structure [130]), which physically tethers mitochondria and ER and facilitates cooperative phospholipid synthesis, intraorganellar calcium-exchange, and mitochondrial DNA inheritance in a coordinated manner [131]. The nuclear control of mitochondrial biogenesis occurs by transcription factors: Nuclear respiratory factor (NRF)-1 and NRF-2. NRFs have a vital role in influencing the expression of genes required for maintenance and function of mitochondrial activity. NRFs also affect the gene transcription and translation machinery, which indirectly affects the production of respiratory subunits encoded by mtDNA (auxiliary factors necessary for promoter recognition (TFB1M, TFB2M), mitochondrial transcription factor A (Tfam), single RNA polymerase (POLRMT), termination factor (mTERF)) [132].

### 3.2. Compartmentalized Signaling

**Figure 5:**
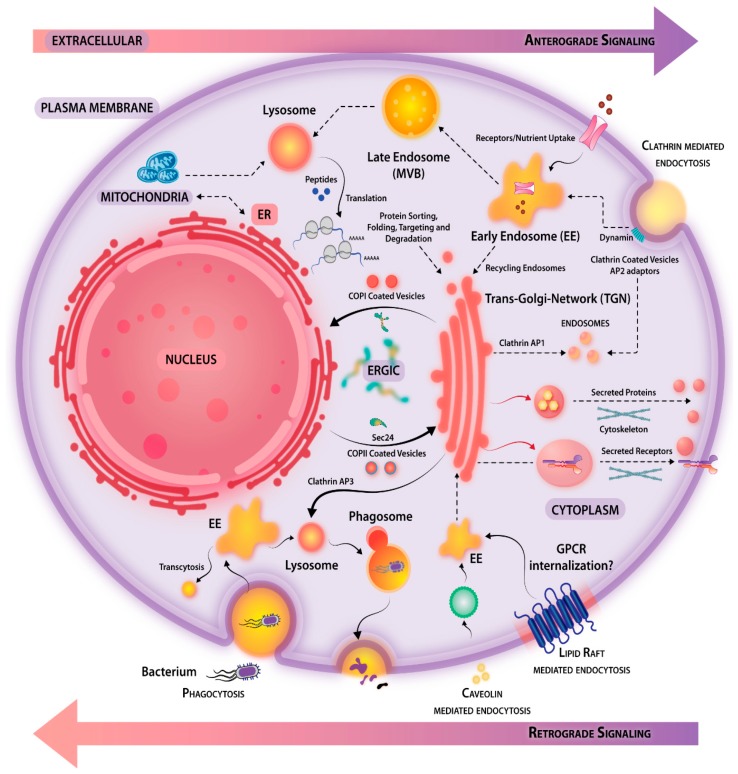
Overview of intracellular vesicular trafficking-cellular organelle rough endoplasmic reticulum (RER) Coat protein I and Coat protein II form vesicles that mediate cargo transport between Golgi to ER and ER to Golgi, respectively. Of the major endocytosis pathways, clathrin-coated vesicles form the early endosomes that mature into late endosomes that subsequently fuse with lysosomes leading to protein recycling. Clathrin, by associating with Adaptor protein (AP) 1 and 2, extends vesicular transport that involves trans-Golgi network. AP3 however, transports proteins to lysosomes and other related organelles. Moreover, receptor endocytosis (GPCR) can occur in clathrin-dynamin-dependent manner. Caveolin also forms endosomal vesicles and joins in the classical endocytic pathway. Phagocytic cells engulf pathogens and effect its lysosomal degradation.

Cells are not an amorphous mixture of biologically active molecules, proteins, carbohydrates, and nucleic acid (DNA and RNA) and their derivatives, bound within lipid-rich enclosures, but comprise of distinct compartments, which developed during the course of evolution to provide specificity to signaling or to generate diversity. Cellular organelles interact by vesicular transport networks (Figure 5). The cargo-containing vesicles are synthesized from a donor compartment with the help of coat proteins (COPI, COPII, clathrin) and adaptors. These vesicles are then transported to acceptors compartments with the help of anchor proteins, where SNAP receptor protein (SNARE)-mediated fusion occurs, resulting in cargo delivery. Vesicular trafficking enables proteins in membrane-bound vesicles to move back and forth from the cell compartments to the plasma membrane. The endocytic compartments containing target cargos move within the cell as observed in the case of RILP (Rab7-interacting lysosomal protein), which assists in the recruitment of dynein–dynactin motor complexes to Rab7 containing endosomes and lysosomes, their targeting towards cell periphery, and timed expulsion [133]. Molecules internalized from the plasma membrane generate endosomes at the trans-Golgi network (TGN) (shown in Figure 5). These molecules either follow the endocytic pathway for their degradation or may be transported to the PM again. Molecules can be transported to endosomes through the TGN, to be tagged for destruction in lysosomes, or recycled back to the Golgi. Using immunoelectron microscopy, Stoorvogel et al. proposed a model for maturation of endosomes [134]. A mechanobiological role of compartmentalization can be observed in case of the polarization of the epithelium [135] where processes protruding from apical and basolateral surfaces can act as a substratum for cellular attachment or may act as a secretory surface for various glands.

### 3.3. Cell Adhesion and Membrane Protrusion

The shape of the cell, protrusions, and retractions of the membrane and adaption to polarization, all are aspects of fine-tuned balancing acts of endo- and exocytosis. Directional cell migration is a fundamental process controlling the biology of development, inflammation, wound healing, metastasis, etc. A key aspect of migration of adherent cells is the formation of focal adhesion (FA) junctions (transient tethering to ECM through integrin clusters). Cell soma is propelled forward by contraction of FA-associated actin stress fibers. The process of FA disassembly may be regulated by components like dynamin, microtubules, and FA kinases [136], and their endocytic turnover occurs in a clathrin-dependent manner [137]. The physical links between the cellular machinery that relate to cell adhesion and membrane protrusions are proteins like vinculin. For example, in response to EGF stimulation, the Actin-related protein 2/actin related protein 3 (Arp2/3) complex is directly recruited to the hinge region of vinculin [138]. Membrane protrusions interact with the extracellular environment, present peptides on the cell surface, and mediate the secretion and engulfment of materials. Membrane protrusions can dynamically adjust polarity axis in conjunction with small GTPases like Rab8 [139]. Dynamin-2 is a well-known protein that mediates clathrin- and caveolin-mediated endocytosis (Figure 5). Dynamin-2 serves as an integral component of signal transduction, surface remodeling, and nutrient scavenging via clathrin-mediated and clathrin-independent endocytosis.

In eukaryotes, the endosomal sorting complex required for transport or ESCRTs are well-studied proteins required for intracellular transport [140]. These proteins are responsible for orchestrated membrane-deformation and membrane-scission events during the cytokinesis phase of cell division [141]. They direct processes like multivesicular bodies (MVB) formation, endocytosis, retroviral budding [142], etc. Specialized structures called MVBs or exosomes are mature endosomal compartments that harbor intraluminal vesicles (ILVs), which are comprised of protein and lipids [143]. These ILVs are frequently degraded by lysosomes. ILVs are produced by invagination of endosomal membranes and sequential action of the ESCRT system. Molecular analysis in diverse eukaryotic organisms reveals that various ESCRT components like ESCRT-I, -II, -III, and ESCRT-III-associated components are found in many eukaryotic subgroups, indicating that some features of ESCRT biology remain conserved within different eukaryotic genera [144]. ESCRT, which are a part of endosomal trafficking, mediates signaling events that involve the translocation of proteins to the nucleus or from one cellular compartment to another. Using an RNA interference (RNAi) approach, the ESCRT machinery and major histocompatibility complex class II (MHC-II) associated proteins were targeted. Subsequently, vesicles for endosome-associated heat shock protein HSP70, MHC-II, tetraspanin CD81, and endosomal tetraspanin CD63 were analyzed. The exosomes secreted by these cells and primary MHC-II-expressing DCs were found to be different in size and proteins [145].

## 4. Complexity in Signaling

Cell signaling is a multifactorial system that represents knot-like schematics of signaling cascades and reflects that none of the pathways in cells operates at isolation. Signaling interplay is inevitable in complex scenarios whereby the system perceives a combination of stimuli (chemokines, cytokines and growth factors, and pathogenic moieties) yet simultaneously maintains the fidelity of signaling output.

### 4.1. Interactions between Pathways

**Figure 6:**
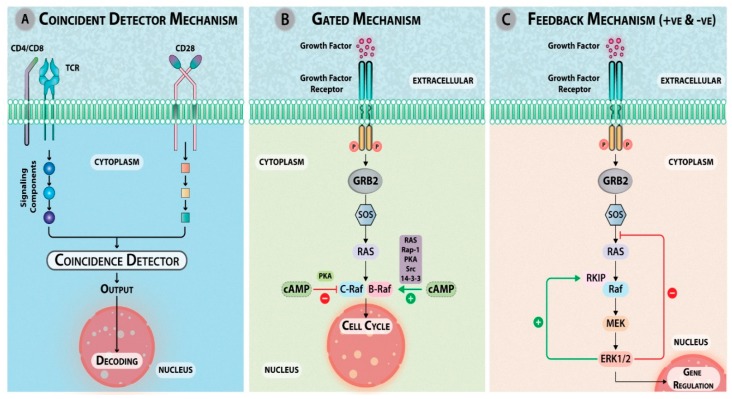
(**A**) In coincident detector mechanism; (**B**) gated mechanism; (**C**) feedback mechanism (positive and negative).

The complexity in the signaling nexus arises due to the interaction or crosstalk between signaling pathways. Many such interaction models have been proposed to understand how, from multiple signaling inputs, signal integration can occur that eventually dictates the biological output. In the “Coincident detector mechanism”, the two pathways 1 and 2 comprised of one or many signaling components converge at a single functional unit called the detector. The detector can recognize the spatiotemporal activation of pathways 1 and 2 and as output can generate a response that could be either synergistic or functionally different from the combinatorial effect of 1 and 2. Such interaction enables a cell to create diversity in terms of output when two or more pathways are activated simultaneously. For example, signaling originating from different cell surface receptors like TCR and costimulatory molecule like CD28 can converge at a common intracytoplasmic coincident detector and can give rise to an output (Figure 6A) [146]. Type-I adenylyl cyclase acts as a detector and allows synergistic regulation of cAMP response element (CRE)-mediated transcription by intracellular Ca^2+^ and isoproterenol and regulate long-term synaptic changes in neurons [147]. Another example is ERK that acts as both a coincident detector and a signal integrator. The simultaneous stimulation of a receptor tyrosine kinase by its ligand and of receptors of the LDL-receptor-related protein (LRP-1) by low-density lipoprotein (LDL) or lactoferrin leads to sustained ERK phosphorylation. Only when two signals are simultaneously present, the threshold is reached [148]. In the “gated mechanism”, pathway 1 is regulated via activation of pathway 2 and the generated response is modified, but not distinct from that on the activation of one pathway exclusively. For example, cAMP acts as a gate in a multitude of cellular responses. Upon growth factor stimulation, Ras GTPases are activated, which associate with and activate their effector Raf, but the effect depends on the cell type and context. cAMP blocks C-Raf through PKA, whereas cAMP can activate B-Raf via PKA, RAS, Rap-1, and Src. In cells like NIH-3T3, C-Raf is negatively regulated by PKA [149], while in PC-12 cells, B-Raf is activated by PKA [150] (Figure 6B) [151,152]. The “feedback mechanism”, which could be a positive feedback or negative feedback, is a modified gated mechanism wherein one initial signal modulates multiple pathways or multiple effectors. Feedback loops serve the purpose of amplifying or attenuating a signal, hence altering the dynamics of the signaling network. As feedback loops connect the output to the input, this regulatory mechanism fine tunes the signaling. Of the multiple effectors, one effector regulates the biological outcome while the other regulates the signal flow to the effector that produces this effect. For example, ERK1/2 positive feedback results in the inactivation of Raf kinase inhibitor protein (RKIP) and thus the activation of Raf, whereas in the negative feedback, it inhibits Grb2–Ras–Guanine nucleotide exchange factor son of sevenless (Sos) complex (Figure 6C) [153]. Another example is the case of light perception by rods and cones in the eye that respond to light via a neural impulse generated by hyperpolarization of membranes mediated by cGMP gated Ca^2+^ and Na^2+^ channels. The activation of the regulatory enzyme phosphodiesterase leads to a decrease in the level of cGMP, the closing of cGMP gated Ca^2+^ channels, and a concomitant decrease in the intracellular Ca^2+^ levels. This leads to a decrease in the activity of Ca^2+^/Calmodulin-dependent adenylyl cyclase. This simultaneous decrease in adenylyl cyclase and activation of phosphodiesterase leads to a decline in cAMP levels and PKA activation, modulating phosducin phosphorylation. The binding of G_βγ_ subunit of transducin is determined by the phosphorylation state of phosducin. Hence, lower activation of phosducin reduces the signal flow from rhodopsin to the cGMP phosphodiesterase [154].

Such a feedback regulatory mechanism also occurs in the mechanistic target of rapamycin (mTOR) pathway where a loop exists from mTOR complex-1 to its downstream ribosomal S6 kinase to insulin/IGF signaling and mTOR complex-2. These proteins regulate many cellular processes by forming two biochemically and functionally distinct complexes mTORC1 and mTORC2 (Figure 3). Under hyperstimulation of growth factor (IGF-1), mTORC1 induces the degradation of its own signaling component insulin receptor substrate 1 (IRS1) [155] that is responsible for activation of the PI3K-Akt pathway and the subsequent activation of mTORC2.

The interactions between signaling pathways are dependent on the molecular diversity of the signaling components. The spatio-temporal sorting of signals can occur in highly organized networks with a multitude of interacting partners [156]. Just as Erwin Schrödinger debated how unpredictable behaviors of individual molecules during diffusion collectively could give rise to a statistically predictable behavior (‘What Is Life? The Physical Aspect of the Living Cell’), the signaling outcome is usually predictable in spite of the involvement of a multitude of signaling molecules. Figure 6 represents different models of cellular interaction.

### 4.2. Post-Translational Modifications (PTMs)

PTMs are reversible or irreversible chemical modifications in proteins following the process of translation. Proteolytic cleavage is one of the fundamental modifications, which adds or removes additional amino acids or a certain portion of protein. Nonetheless, in eukaryotes, the spectrum of PTMs extends to acetylation, amidation, biotinylation, farnesylation, formylation, geranylgeranylation, glycation, glycosylation, hydroxylation, methylation, mono/poly-ADP-ribosylation, myristoylation, oxidation, palmitoylation, SUMOylation, and phosphorylation. Ubiquitination is the most crucial PTM in eukaryotes since it governs the protein turnover in the cell by acting as a proteasomal degradation signal. More so, specific signals (degrons) can link a range of protein PTMs directly to the ubiquitin-proteasome system (UPS) [157] and thereby regulate protein turnover. Neddylation is a PTM in which ubiquitin-like protein NEDD8 is conjugated to its target proteins, comparable to ubiquitination, but employs its own E1 and E2 enzymes [158].

With the advent of phosphoproteomics in the 21st century, PTMs are widely studied and more than 40 PTMs have been identified to be associated with diseases such as cancer, autoimmunity, and neurological defects. The PTMs have unequivocal roles in dictating the protein’s stability and its functions. PTMs are also considered to be important in the maintenance of circadian clocks. A study by Johnson et al. has elucidated the endogenous circadian system in *Cyanobacteria*, that exert ‘pervasive control’ over cellular processes including global gene expression [159]. The complexity of phosphorylation can be studied in both bacterial and mammalian systems. The construction of protein–tyrosine phosphorylation interaction networks can reveal novel substrates, kinase cross-talks, and kinase activators [160]. Just like other PTMs, phosphorylation allows rapid and reversible changes of the factors like a protein’s enzymatic activity, interaction with partners, oligomeric state, cellular localization, half-life, and its turnover. In a protein, amino acid residues like serine, threonine, and tyrosine are the vulnerable targets of phosphorylation. Conformational changes as a result of a PTM in general, may affect the overall protein distribution in the cytosol as these changes may reveal sequestered NLS (nuclear localization signals) on certain transcription factors [161]. Similarly, tyrosine kinases are often autophosphorylated within their dimeric catalytic cores and this potentiates their RTK activity. PTMs like tyrosine phosphorylation may also create specific binding sites for adaptor recruitment such as those harboring Src homology domains. Such a case is exemplified by TRANCE (TNF-related activation-induced cytokine) which is capable of activating antiapoptotic serine/threonine kinase Akt/PKB through a signaling complex through c-Src and TRAF6 [162]. PTMs play a pivotal role in the diversification of proteins and single protein can function diversely in different cell and tissues types. Nonetheless, it also serves as a way for the cells to modulate the turnover and functions of proteins and to generate heterogeneity. This can be further elucidated in the context of biological oscillation of certain proteins that control the circadian rhythm. For example, in class *Chordata* (*Mus and Homo*), brain and muscle ARNT-Like 1 (BAML1) and CLOCK proteins shuttle between the cytoplasm and nucleus [163], suggesting the role of PTMs in regulating the mammalian circadian clocks [164].

### 4.3. Engagement of Different Signaling Modules

The multi-tier complexity in signaling events is attributable to compartmentalized signaling, distinct pools of signaling components, and their spatiotemporal expression and activation. As most of the signaling components are tethered to the plasma membrane, it is the hub of cellular signaling. Another platform for signaling are the cellular organelle, each with its distinct biochemical microenvironment. In addition to these fixed pools of signaling complexes within the organelle and certain translocating molecules [165] that shuttle between different parts of the cell, dynamic signalosomes in the form of vesicle-bound nano-clusters also exist [166]. Cellular scaffolds like the cytoskeleton makes an assembly line with signaling components arranged in an orderly fashion to relay a signal [167], or in some cases, the signaling enzymes are physically linked as a giant multi-enzyme complex as in polyketide synthesis [168].

During the course of signaling, different signaling modules may be engaged that play a decisive role in the signaling outcome. For instance, *Drosophila* Frizzled (Fz) receptor’s affinity for the Wnt ligand decides the signaling through two biochemically and physiologically distinct pathways. One through the canonical Wnt pathway to regulate gene expression and another through Rho GTPase and JNK for cytoskeletal reorganization [169]. Secondly, a single receptor can activate multiple transducers in different tissues of the same organism. For example, in *Caenorhabditis elegans,* upon activation of receptor tyrosine kinase (RTKs), the signal is relayed through IP_3_ in the gonadal tissue while through Ras-MAPKs in the vulva and other tissues [170]. Thirdly, multiple signaling pathways can converge at one focal node and coordinate the regulation of certain genes. For example, the cytokine-induced C-C chemokine monocyte chemoattractant protein-1 (MCP-1) is regulated by both p38- and Phosphatidylcholine-specific phospholipase (PC-PLC)-dependent pathways [171]. Fourthly, different cell types can respond differently to the same stimulus owing to the presence of cell-type specific factors. For example, the cell-type specific response to TGF-β signaling is determined via binding of Smad2/3 protein with a class of cell-type specific master transcription factors [172]. Moreover, the presence of certain extracellular and transmembrane signaling antagonists and activators can also influence cellular responsiveness. Wnt signaling can be inhibited by extracellular factors like Cerberus, Frizzled-related proteins (sFRPs), Dickkopf proteins (Dkks), Wnt-inhibitory factor 1 (WIF-1), Sclerostin domain-containing 1 (Wise/SOST), insulin-like growth factor binding protein 4 (IGFBP-4), and by transmembrane factors like adenomatosis polyposis coli down-regulated 1 (APCDD), Wnt-activated inhibitory factor 1 (Waif1/5T4), while activated by R-spondins and Norrin [173]. Finally, compartmentalized signaling from spatially distinct centers can further contribute to the complexity and specificity of signaling. Organellar specific pools of MAPKs (e.g., within Golgi) can have digital (where signaling output rapidly switches between an ‘ALL or HIGH’ or ON mode and a ‘NO or LOW’ or OFF mode signaling) or analog (graded transformation of signaling) signaling for different modules [174].

## 5. Translational Value of Understanding Signal Transduction

Defects in signaling pathways and/or pathogenic perturbation can cause a number of diseases. Pathogens can interfere with signaling intermediates or can phenotypically or genotypically modify signalosomes. For a given signalosome, a defined operational range of stimulus strength exists, over or under which its functioning is hampered. Pathogens can remodel the components of a signalosome leading to its hyperactive or hypo-sensitive altered states. The effect of receptor clustering can also play a decisive role in infection outcome. For instance, in CD40 signaling, a low signal dose activates ERK1/2 while a high dose activates p38MAPK with the production of IL-10 and IL-12, respectively, that determines the course of *Leishmania* infection [175]. Certain virulence factors (peptides and small molecules) produced by bacteria target key signaling intermediates like GTPases, kinases, and other cellular regulatory components within the host. Two well-elucidated examples are the pathophysiology of cholera and pertussis. *Vibrio cholerae* produces the cholera toxin that ADP-ribosylates the α_s_ subunit of heteromeric G_S_ proteins, rendering it constitutively active and hence activating adenylyl cyclase. This leads to elevated levels of cyclic AMP in the intestine that affects the efflux of chloride ions and water, causing dysentery [176]. In the case of *Bordetella pertussis* toxin, the α_i_ subunit of the heterotrimeric G_i_ protein is targeted and converted into a GDP-bound state so that it cannot regulate downstream production of cyclic AMP. Excessive cellular ATP is converted to AMP leading to inhibition of cell signaling pathways and hampering the phagocytic response to infection [177].

Signaling components are being identified and targeted for the generation of small inhibitory molecules and antibodies. The fundamental step in drug designing is the identification of the appropriate target, followed by evaluating the druggability of the signaling components, and its role in a particular disease. In the case of diseases like cancer, multiple signaling components of the same pathway can be lucrative drug targets. For instance, Ras, the small cellular GTPase, has been identified as a drug target by various studies owing to its role in different types of cancers. Post-translational modifications in Ras are important for its membrane targeting, hence several competitive inhibitors of the enzyme Farnesyltransferase (FTase) like R115777 and SCH55335 are under clinical evaluation in patients with solid tumors and refractory acute leukemia (De Bono, 2002). As FTase inhibitors are active in blocking the isoform H-Ras, but not K-Ras or N-Ras, alternatively, Geranylgeranyl transferase inhibitors (GGT) were also developed. In addition to this, several antisense oligomers are in various phases of clinical trials [178]. Downstream to Ras are signaling proteins having Ras-binding domains (RBDs) like Raf, which further phosphorylate MEK. Mutations in Raf and a constitutively active MEK have been identified in different tumor types. Hence, Raf and MEK are also potential drug targets against cancer. Inhibitors of Raf, like ZM336372, that exhibited anti-cancer activity and bis-aryl urea (BAY 43-9006) is in the clinical trials [179]. Small molecular inhibitors of MEK like PD09059 [180] and PD184352 [181] were tested in clinical trials. PKCs and Bcl-2 can also activate Raf, demonstrating how various signaling components of the RTK pathway can be drug targets [182]. ISIS 5132, an antisense oligonucleotide targeting PKCs [183], and G3139 [184], a phosphorothioate antisense targeting Bcl-2, were clinically evaluated.

The organization of a protein into distinctive modules of signaling forms the very fundamental basis of their therapeutic targeting. For example, there are domain-specific inhibitors of mTOR kinases known as Rapalogs, that target the FKBP-rapamycin-binding (FRB) domain [185]. However, in case of drug-resistant cancer patients with activating mTOR mutations, for those who acquire resistance to first generation inhibitors of mTOR, a second generation of inhibitors that targets the kinase domain (TORKIs) has been developed. In situations where both first and second generation fail to show chemotherapeutic benefits, a third generation of inhibitors is used, which is based on the unique juxtaposition of each drug-binding pocket to design a bivalent interaction. This allows the chemo targeting of resistant mutants [186]. Thus, identification of small molecules that can mimic a particular substrate and thereby occupy the ligand-binding domain in an enzyme may serve as an alternative strategy for pharmacological targeting of important proteins. Similarly, genome-editing systems using CRISPR/Cas9 can be used to mutate the gene regions encoding particular protein domains. This could result in a new and even more efficient method to screen for protein targets that are druggable and critical to the survival of cancer cells [187]. As mentioned earlier the PI3K/Akt/mTOR pathway is hyperactivated in many cancers, moreover, mTORC1 and ribosomal S6 kinase exert negative feedback to suppress hyperstimulation by growth factors. mTOR inhibitors failed as an anti-cancer drug due to suppression of the negative feedback loops from mTOR and S6, hence reversing the anti-proliferative effects of the inhibitor. Thus, a better understanding of the feedback loops would enable the designing of therapeutics with better efficacy [188].

In addition to growth factor receptor, Janus kinase and Src kinase can activate the cytoplasmic proteins Signal transducers and activators of transcription (STAT). STATs, mainly STAT-3 and STAT-5, that are associated with various forms of malignancies are potential drug targets. Not only the growth factor receptor pathway, but also defects in the Wnt signaling pathway, mainly β-catenin, represent an appropriate drug target in Wnt-signaling associated cancers [189]. Clinical trials are ongoing using small molecule-targeting protein kinase D, which is involved in pancreatic cancer [190], and insulin-like growth factor-1 receptor (IGF-1R) that is involved in MAPK and PI3K/Akt pathways and also cross-talks with EGFR pathway. Monoclonal antibodies (mAb) against IGF-1R like Cixutumumab, Figitumumab, Dalotuzumab, R1507, and Ganitumab are in different phases of clinical trials [191]. Trastuzumab, an mAb, which blocks HER2 overexpressed in invasive breast cancers underwent two axial trails in patients with metastatic breast cancer [192]. In the treatment of metastatic colon cancer, Bevacizumab in combination with irinotecan, fluorouracil, and leucovorin that acts on VEGFA (angiogenesis pathway), gained the approval of FDA as the first systemic anti-angiogenic drug [193]. TLN-4601, a farnesylated dibenzodiazepinone that blocks the Ras-MAPK pathway and accumulates in gliomas by binding to benzodiazepine, is another candidate for treating glioblastoma and has undergone phase II trials [194]. Another approach being used is to combine small-molecule inhibitors of MEK (PD0325901) and Akt (API-2) with radiotherapy for treatment of pancreatic cancer. A comprehensive understanding of the signaling pathways enables the identification of critical or central nodes within a signaling network. Targeting such nodes would help in designing therapeutics with higher efficacy. For example, PTPN11 is the central node in RTKs, activated in the Ras-MEK-ERK pathway. Cells may acquire resistance to cancer drugs through activation of RTKs, which can be inhibited by selective targeting of PTPN11 [195]. This therapeutic approach of targeting signaling molecules has fewer side effects, but a thorough validation of the efficacy and toxicity should be done in a clinical setting.

## 6. An Evolutionary Perspective of Signaling

Life started with unicellular forms having a limited number of genes and proteins. As a result, cell signaling was less complex. With the evolution of multi-cellular life forms, the number of genes has increased, new signaling intermediates have been added, and the signaling networks have become more complicated. This addition of a novel array of signaling proteins contributes to diverse responses to the same stimuli across different phyla. Complex domain architecture of proteins in animals when compared to plants and lower organisms enables the ramification of the signal transduction process through different proteins. Genome studies in *Protists* have revealed a great variety in the genome in terms of regulatory proteins it encodes for, implying the role of signaling protein in determining diversity in eukaryotes [196]. Homologs of core components of Wnt, Notch, Hedgehog, receptor-tyrosine kinase, JAK-STAT, and TGF-β signaling were found in *Poriferans* like *Oscarella carmela* [197]. These components are present in eumetazoans from *Cnidaria* to vertebrates. Signaling intermediates like small cellular GTPase Ras [198], phosphoserine/threonine-binding dimer adaptor protein 14-3-3 [199], and cAMP-dependent protein kinase were found in protozoans *Plasmodium falciparum*, *Plasmodium yoelii*, [200], etc. The appearance of receptor tyrosine kinases predates the evolution of multicellular organisms [201].

Communication might have played a decisive role in the course of evolutionary survival of primitive prokaryotes; quorum sensing in prokaryotes is one such evidence. Quorum sensing evolved as one of those basic mechanisms where cellular density-dependence dictates the natural ecological structuring in bacterial populations. Within microbial communities, it helps recapitulate stabilization or destabilization with respect to environmental cues like fluctuation in population density. An example such as quorum sensing in Gram-positive and Gram-negative bacteria illustrates how signals turn on cellular communication circuits and travel from ‘outside to inside’ to regulate a diverse array of physiological activities like production of antibiotic, competence, conjugation, induction of dominance, symbiosis, virulence, etc. [202]. Interestingly, in Metazoans, major aspects of biology that are controlled by signal transduction are growth, reproduction, and stimuli-triggered motor responses and involuntary reflexes at organismal level. Cellular metabolism, changes in gene regulatory protein, altered cytoskeletal changes for forming protrusions, motility, stress responses, cell division, or differentiation are some facets of signaling regulated cellular changes.

There are multiple theories regarding the evolution of the receptor–ligand pair. According to one, the receptor and the ligand (encoded message) evolved separately as cell signaling did not evolve as a stimulus–response circuit with definitive output but rather by chance as a trial–error method with infinite possible outcomes. By gene transfer between species, the pathways were transmitted, inherited, and a few became obsolete owing to the deletion of signaling components. For example, Agouti signaling protein (ASIP), an endogenous antagonist of melanocortin receptor that regulates various physiological traits in primates, was deleted in the genome of gibbons [203].

Contrary to this, the co-evolution theory of ligand–receptor states that the receptor and its ligands co-evolved. One such example is gonadotropin and its receptor. Primordial gonadotropin β-subunit underwent gene duplication and the residues were replaced with those that afforded specificity in binding, leading to the formation of Luteinizing hormone (LH) and Follicle stimulating hormone (FSH). These residues, if replaced, gives rise to a chimeric universal hormone with no specificity. In the case of the receptor, the ancestral receptor gene following gene replication underwent a mutation that introduced specific determinants in various regions of the receptor. This process introduced the element of specificity to the receptor. Hence, continuous refinement of specificity occurred in the structure of both the ligand and the receptor [204]. Others state that from prokaryotes to higher organisms, the same messengers are used and the diversity arises due to the differences in the magnitude and spatiotemporal location of the messengers, their respective receptors, and effectors [205]. The receptors arose devoid of the ligand, and the receptor–ligand interaction occurred later in evolution [206]. Thus, in a Darwinian process under natural selection, incremental evolutionary changes contributed to the complexity of cellular signaling [207]. Because certain pathways are inevitable for the reproduction and survival of the organism like the Gonadotropin pathway, the receptor–ligand evolution in such cases would be tightly coupled. Experiments that demonstrate cross-species binding of receptor–ligand reinstate the fact that specificity of this interaction is low. For example, chicken Luteinizing hormone (LH) was found to bind the rat LH receptor with a higher affinity than rat LH [208]. Molecules having hormone-like action appeared early in evolution. For example, substances chemically resembling thyroid were discovered in lower invertebrates, algae, and sponges. GABA_B_ and Frizzled-like receptors have been detected in *Dictyostelium discoideum* [209], EGFR-like and insulin-like receptors in sponges [210], and mammalian ionotropic glutamate-like receptor evolutionarily appeared in plants like *Arabidopsis* [211]. With the evolution of the receptor, concomitant evolution of the process of the relay of information occurred. Although signaling pathways have evolved into a complex mesh, with multitudes of pathways and numerous interacting partners, biochemical and genetic studies have revealed that a select few conserved pathways are sufficient to give rise to cellular and morphological diversity. The specificity of these pathways is based on factors like the structural, biochemical, and biophysical properties of a cell type and the cross-talks between various signaling pathways. Throughout the process of animal development, majorly, seven conserved pathways play a crucial role [212]. These are namely the receptor tyrosine kinase pathway (RTK), Janus kinase/signal transducer and activator of transcription (JAK/STAT) pathway, transforming growth factor beta (TGF-β) pathway, notch pathway, hedgehog (Hh) pathway, wingless-related (Wnt) pathway, and nuclear hormone pathway [213,214,215,216,217,218]. Since these seven conserved pathways are sufficient to pattern diverse outcomes, it implies the redundancy of signaling intermediates and plasticity of signaling pathways. In addition to this, toll-like receptor pathways have certain evolutionarily conserved regulatory proteins called evolutionarily conserved signaling intermediate in toll pathways (ECSIT) that serve as signaling adaptors. ECSIT proteins interact with TRAF6 and MEKK1, thereby activating transcription factors like NF-κB and AP-1 [219]. Tumor necrosis factor (TNF) that emerged during the Precambrian era has certain evolutionarily conserved primary or conformational structures that play a role in cell death [220].

## 7. Conclusion: Past, Present, and Future of Cell Signaling

### 7.1. Major Discoveries in the Field of Signal Transduction

Many path-breaking discoveries have shaped the current understanding of cell-signaling networks. The major discoveries that laid the foundation and facilitated the advancement of cell signaling research are compiled in Table 2.

### 7.2. Current Methods in Cell Signaling

Cell signaling studies have been transformed by continuously evolving molecular biology methods, microscopy, mathematical, and statistical methods to process the data and spectrophotometric methods. A widely used approach refers to isolating an individual cell followed by in vitro studies to evaluate the effects of specific signals/chemicals on cellular responses; whereas a more inclusive method involves studying the signaling events in vivo. Major approaches and techniques devised to study cell signaling are provided in Table 3.

#### Mathematical Modeling, Systems Biology, and –OMICS

The –OMICS refers to collective technologies used to explore the functions, and interactions amongst cellular molecules and includes genomics, proteomics, metabolomics, transcriptomics, glycomics, and lipidomics. High-throughput methods in genomics have provided important insights into cell signaling networks. For example, signal transduction networks (STNs) can be uncovered by integrating protein interaction with gene expression data using an integer linear programming model [221]. The yeast MAPK pathway has been extensively modeled to delineate the order of signaling components in the pathway [222]. As signaling networks evolve from simple to complex ones, the signaling directionality is scattered across various signaling nodes due to multiple protein–protein interactions. Alternatively, mathematical modeling can help us understand the complexity of the signaling networks better and, thus, may help in the restoration of directionality in signaling and understanding the topologies of signaling network. Logical modeling formulas including Boolean, fuzzy logic, and differential equations-based methods can be implemented to capture quantitative data [223,224,225]. A unison between computational modeling and the use of predefined signaling motifs [226,227] explains the aforementioned premise. Using a proteomics-based approach, a new method for interpreting complex phosphorylation data in the context of protein–protein interaction networks is developed. Such methods work to identify active proteins and pathways and pinpoint putative phosphosites. Using this approach EGFR and insulin signaling datasets were analyzed [228]. A similar approach is adopted to gain a better understanding of skeletal muscle proteomics, in the context of insulin resistance and exercise biology [229].

Systems biology and protein engineering has enabled modeling of cellular properties simultaneously, fostering the fact that cells are dynamic systems and the phenomena like feedback–feedforward regulations [227] in signaling pathways are predominant in governing a cell’s behavior. Furthermore, large-scale system integration allows designing pathways de-novo, which also broadens our current understanding of signal transduction [118]. In order to construct large-scale protein interaction networks (PINs), knowledge of gene expression, transcriptional control, PTMs, protein turnover, and functioning of a protein in a time-dependent manner is a prerequisite. Such a functional map (PINs) may also elucidate the role of the second messenger cyclic diguanylate (c-di-GMP) in the pathogenesis of microorganisms e.g., *Mycobacterium tuberculosis (Mtb).* Quorum sensing pathways associated with pathogenesis, biofilm formation, and acquisition of persistent drug-resistance characteristic can be attributed to c-di-GMP [230]. Similarly, *Mtb*-human protein–protein interaction maps may identify a switch between host anti-pathogenic responses. Such maps are constructed ab initio using mass spectrometry-based approaches [231,232]. Other techniques employed for studying protein interaction networks are two-hybrid systems [233], analysis of genomic sequences [234], structure-based elucidation [235], and are theoretically performed by machine learning [236]. Penn et al. identified a novel *Mtb* virulence factor that associates with the host ubiquitin ligase, CBL [237]. High-throughput liquid chromatography and mass-spectrometric analysis have allowed a priori quantification of small metabolites within a cell and the ability to deduce their biochemical fates. In *Mtb,* the central carbon metabolism (CCM) became of great interest in recent times [238]. This prompted unique designing of the metabolic pathways associated with virulent strains of *Mtb*. High-throughput screening can also help pharmacologists to develop intervening compounds that interfere with *Mtb’s* CCM, and at the same time, have minimal collateral effects on the host.

Again, considering the promiscuity of many proteins interactions and cross-regulation between signaling pathways, system engineering becomes a daunting task. This could be minimized using protein homology modeling since many protein sequences are evolutionarily conserved and, thus, can be classified into different families. Protein that belongs to the same family frequently shares similar 3D-structure and notable similar attributes, which allows a structural deduction of all proteins in a family, based on one common member. This evolutionary relationship lay grounds for structural genomics and proteomics; a large initiative towards structural characterization of most proteins where a representative protein from the family is structurally solved and the structures of others are reliably predicted with homology modeling [239]. Pfam database currently annotates 16,712 families and 604 clans (Pfam as on 31 September 2018, 17,929 entries) [39]. If a sequence identity cutoff ~30% is applied (a generalized number for successful homology modeling), the statistics predict about 10,000–30,000 of all proteins in nature [240]. Another level of complexity in pathway engineering is modulating the signaling output via manipulation of targeting signals to control protein subcellular localization, oligomerization, and proteolysis [241]. The lack of a priori knowledge in the context of localization signals, protein folding, PTMs, degradation mechanisms, thermodynamic behavior, protein turnover, spatiotemporal expression of proteins, and isoform-specific functions of proteins are some challenges faced in an automated signal-transduction engineering exercise. Beyond these challenging broad biological issues, the efficient design of signaling pathways is also limited by the availability of suitable linkers when shuffling protein domains [242]. The inherent stochastic variations in which noise and signal propagation occur along the transmembrane or in endocytic compartments, also affects signaling and is challenging to engineer [243]. Similarly, multidomain proteins [38] with rewired functionalities change the input–output behavior of the modeled system [244]. Nearly seventy-five percent (75%) of the eukaryotic proteome consists of multidomain proteins [245]. The aggregation behavior of multidomain proteins has already been reported [246,247]. Oligomeric protein assemblies can give rise to diverse functionalities in different organisms. These underline some most revolutionary concepts like protein-based genetic elements [248], membrane-free compartmentalization, evolutionary capacitance [249], and evolution of cryptic genetic variations [250]. Under these cases, typically the oligomers or aggregates results in expression of new phenotypes not previously observed because of their soluble conformations. For example, a prionic conformation of cytoplasmic polyadenylation element binding protein (CPEB) exhibits increased affinity for RNA, contributing to long-term memory formation in metazoans [251]. It has also been observed that preceding viral infection, the human mitochondrial protein (MAVS) forms functional prion-like polymers for activating a highly sensitive and robust mechanism of the immune response [252]. The emergence of such unique functionality of prionic proteins can vary the outcome of modeled signaling greatly. Therefore, while modeling signaling, one must take care of such unique functionality amongst the cellular automata to achieve reproducible results and minimize the unpredictable behavior of the modeled system. Improving algorithms that carry out protein designing and predict large conformation changes in proteins during a variety of physiological conditions like stress is necessary for successful pathway engineering. The design of optimized selections systems with directed evolution of proteins seems to be a legitimate approach towards successful engineering of pathways [253]. A successful designing approach can only be applied to engineer signal transduction if the following prerequisites are met: (1) Correctly identified protein functions; (2) domains, interactive partners that show iterative predictable behavior and allow different permutations, and combinations should exist, also minimizing the noise [254]; and (3) in this process, one may incorporate permissive substitutions in the evolution of receptor specificity [255].

The successful engineering of signaling pathways would be greatly influenced by how scrutinized and robust the engineered models are in mimicking the in vivo behavior of cells. The minimization of context dependence should also be taken care of (as the nature of signaling in neuron is different from that in an immune cell, muscle cells, or in a pancreatic β cell). Finally, manipulation of kinome for phosphorylation may greatly influence the outcome of the system and uncovering new phosphorylation targets may bring the novelty and stratify behavior of the modeled system [256]. The waves of second messengers [Ca^2+^, cAMP, Guanosine pentaphosphate (P)ppGpp] and status of methylation and acetylation can also affect the engineered signaling outcomes. Synthetically imposing regulation on kinome may provide a viable strategy to enhance signaling. For example, the behavior of Ste5 in yeast gives us information on how multisite phosphorylation can be read out via bulk electrostatic effects to yield a powerful biochemical switch [257].

### 7.3. Future of Cell Signaling

Studies on cell signaling explain how cells sense and respond to the environmental cues, adapt, and coordinate with other components within the system to maintain homeostasis. While the genomic studies identified the organization and expression of the genes that influence cell signaling, the complex functional relationships between the signaling intermediates or their configurations into multi-strata signaling networks remain an enigma [256,258]. Striking similarities between electronic circuits and cellular automata in terms of the existence of hierarchies, topologies, modularity, redundancy, feedback, and feed-forward motifs [259] led to the directed development of computational algorithms to mimic signal outputs. Such experimental and computational data are integrated to develop models of signaling networks [260]. However, these networks are self-restricted and often lack direction, which can be otherwise provided by the development of inter-organellar or compartmentalized signaling as a function of time. Such networks customized in the forms of synthetic genetic logic circuits are now used to predict and manipulate cellular signaling behaviors and have promising applications in areas like biocomputing, environmental biology, biotechnology, and biomedicine [261].

## Figures and Tables

**Table 1:** Summarizes some protein that regulate calcium signaling in diverse cell types.

PROTEIN	CELLULAR/PHYSIOLOGICAL FUNCTION	REFERENCE
***Adenylyl cyclase*** ***(AC Type-1)***	Act as second messengers in regulatory processes in the central nervous system.	[79]
***Annexins***	Annexin I modulates cell functions by controlling intracellular Ca^2+^ release.	[80]
***Ca^2+^/Calmodulin-dependent protein kinase (CaMK)***	Wnt7a signaling promotes dendritic spine growth and synaptic strength through Ca^2+^/Calmodulin-dependent protein kinase II.	[81]
***Ca^2+^-ATPase*** ***(SERCA)***	The sarco/endoplasmic reticulum Ca^2+^ ATPase (SERCA) is the third element in capacitative calcium entry.	[82]
***Ca^2+^-dependent endonucleases***	Ca^2+^/Mg^2+^-dependent endonuclease are drivers of apoptosis.	[83]
***Calcineurin***	Calcineurin is a common target of cyclophilin-cyclosporin A and FKBP-FK506 complexes.	[84]
***Calcium channel blockers***	Important roles in arterial and pulmonary hypertension.	[85]
***Calcium Release-Activated Channel (CRAC)***	STIM1 is a Ca^2+^ sensor that activates CRAC channels and migrates from the Ca^2+^ store to the plasma membrane.	[86]
***Calpain***	Widespread activation of calcium-activated neutral proteinase (calpain) in the brain in Alzheimer disease.	[87]
***Calmodulin***	Calmodulin—an intracellular calcium receptor.	[88]
***Calretinin***	Calretinin: a gene for a novel calcium-binding protein expressed principally in neurons.	[89]
***Gelsolin***	Gelsolin: calcium-and polyphosphoinositide-regulated actin-modulating protein.	[90]
***InsP_3_ receptors***	Inositol 1, 4, 5-trisphosphate (InsP_3_) and calcium interact to increase the dynamic range of InsP_3_ receptor-dependent calcium signaling.	[91]
***NCS-1***	Ca^2+^ signaling via the neuronal calcium sensor-1 regulates associative learning and memory in *C. elegans*.	[92]
***Nitric oxide*** ***synthase (NOS)***	Inducible isoforms of cyclooxygenase and nitric oxide synthase in inflammation.	[93]
***Phospholipase A_2_***	cPLA2 requires calcium for its activity.	[94]
***Phosphorylase kinase***	Direct activation of calcium-activated, phospholipid-dependent protein kinase by tumor-promoting phorbol esters.	[95]
***Protein kinase C***	Protein kinase C as a molecular machine for decoding calcium and diacylglycerol signals.	[96]
***Ryanodine receptors***	FKBP12.6 deficiency and defective calcium release channel (ryanodine receptor) function linked to exercise-induced sudden cardiac death.	[97]
***S100 proteins***	S100: A multigenic family of calcium-modulated proteins containing EF hand motif having intracellular and extracellular functional roles.	[98]
***Synaptotagmin***	Synaptotagmin: A calcium sensor on the synaptic vesicle surface.	[99]

**Table 2:** Timeline of major discoveries in the field of cell signaling.

YEAR	DISCOVERY
*1600*	Robert Hooke 1653/Antony Van Leuwenhoek 1682 first observed cell-like structures.
*1833*	Anselme Payen isolates first enzyme, diastase.
*1839*	Matthias Jakob Schleiden and Theodor Schwann proposed the ‘Cell Theory’.
*1876*	Franz Christian Boll discovered Rhodopsin.
*1878*	W. F. Kühne proposed the theory of Visual transduction, coined the term “enzyme”.
*1884*	J. L. W. Thudichum discovered Sphingolipids (SLs) in the brain.
*1889*	G. Yeo coined the term Protoplasm.
*1894*	F. A. Locke observed that removal of calcium from frog sartorius-muscle preparation could block the transmission of impulses at the neuromuscular junction.
*1895*	N. Cybulski first isolated Adrenaline.
*1903*	E. A. Schaefer introduced “Chalones” hormone-like substance.
*1905*	J. N. Langley proposed the concept of receptive substance and introduced the “Receptor Theory of Drug Action.”
*1905*	Ernest Starling discovered Hormones.
*1906*	Camillo Golgi and Santiago Ramón Y Cajal were awarded The Nobel Prize in Physiology or Medicine for their work on the organization of the nervous system.
*1921*	Otto Loewi discovered Acetylcholine.
*1922*	Frederick Banting and Charles Best discovered Insulin.
*1923*	Frederick Grant Banting and John James Rickard Macleod were awarded The Nobel Prize in Physiology or Medicine.
*1924*	Hans Spemann and Hilde Mangold identified “Spemann’s Organizer” and The Nobel Prize in Physiology or Medicine in 1935 was awarded to Hans Spemann.
*1928*	H. Pollack gave first evidence of Calcium signal.
*1929*	Walter Bradford Cannon described ‘Fight’ or ‘Flight’ responses.
*1935*	Vittorio Erspamer showed an extract from Enterochromaffin cells made the intestines contract.
*1936*	Sir Henry Hallett Dale and Otto Loewi were awarded The Nobel Prize in Physiology or Medicine “for their discoveries relating to chemical transmission of nerve impulses.”
*1943*	Takeo Kamada and Haruo Kinoshita showed Ca^2+^ ions upon injection cause a contraction in muscles.
*1947*	Carl Cori and Gerty Cori were awarded The Nobel Prize in Physiology or Medicine “for their discovery of the course of the catalytic conversion of glycogen.” Along with Bernardo Houssay “for his discovery of the part played by the hormone of the anterior pituitary lobe in the metabolism of sugar.”
*1948*	Maurice M. Rapport, Arda Green, and Irvine Page co-discovered Serotonin.
*1948*	Raymond P. Ahlquist identified subtypes of adrenoreceptors.
*1948*	Vittorio Erspamer discovered Octopamine in the salivary glands of the octopus.
*1953*	Lowell and Mabel Hokin reported the involvement of Inositol-containing phospholipids in cell regulation.
*1953*	Betty M. Twarog and Irvine Page first reported serotonin to be present in the mammalian brain.
*1953*	G. H. Sloane Stanley reported Phospholipase C (PLC) in the mammalian brain.
*1954*	Takashi Hayashi described a special role of Glutamate in electrophysiological processes.
*1955*	Edmond H. Fischer and Edwin G. Krebs discovered the role of Phosphorylation.
*1957*	Lord Todd was awarded The Nobel Prize in Chemistry “for his work on nucleotides and nucleotide co-enzymes.”
*1957*	T. W. Rall and coworkers discovered cAMP.
*1958*	Frederick Sanger was awarded The Nobel Prize in Chemistry “for his work on the structure of proteins, especially that of Insulin.”
*1958*	George Beadle and Edward Tatum were awarded The Nobel Prize in Physiology or Medicine “for their discovery that genes act by regulating definite chemical events.”
*1961*	E. Essner and Alex B. Novikoff discovered Acid phosphatase inside lysosome using electron microscopy.
*1961*	Georg von Békésy was awarded The Nobel Prize in Physiology or Medicine “for his discoveries of the physical mechanism of stimulation within the cochlea.”
*1962*	Max F. Perutz and John C. Kendrew were awarded The Nobel Prize in Chemistry “for their studies of the structures of globular proteins.”
*1962*	R. W. Butcher and E. W Sutherland discovered Phosphodiesterase (PDE) enzyme that removes cAMP.
*1963*	Sir John C. Eccles, Alan L. Hodgkin, and Andrew F. Huxley were awarded The Nobel Prize in Physiology or Medicine “for their discoveries concerning the ionic mechanisms involved in excitation and inhibition in the peripheral and central portions of the nerve cell membrane.”
*1963*	Z. A. Cohn concluded lysosomes act as cells digestive system to recycle compounds.
*1964*	Jennifer Harvey discovered monomeric G-Protein/GTPase in rat sarcoma (Harvey-Ras).
*1964*	Richard A. Lockshin and Carroll M. Williams reported on Programmed Cell Death “Endocrine potentiation of the breakdown of the intersegmental muscles of silkmoths.”
*1965*	G. Heppner and D. W. Weiss Discovered TLR4 as a receptor for LPS.
*1965*	François Jacob, André Lwoff, and Jacques Monod were awarded The Nobel Prize in Physiology or Medicine “for their discoveries concerning genetic control of enzyme and virus synthesis.”
*1967*	Ragnar Granit, Haldan K. Hartline, and George Wald were awarded The Nobel Prize in Physiology or Medicine “for their discoveries concerning the primary physiological and chemical visual processes in the eye”.
*1967*	W. H. Kirsten and L. A. Mayer discovered Kirsten Murine Sarcoma Virus.
*1968*	S. Ebashi and M. Endo discovered Troponin.
*1968*	D. A. Walsh et al., showed that cAMP controlled the activity of PKA.
*1970*	Jacques Benveniste et al., discovered Platelet-activating factor.
*1970*	Luis F. Leloir was awarded The Nobel Prize in Chemistry “for his discovery of sugar nucleotides and their role in the biosynthesis of carbohydrates.”
*1970*	Sir Bernard Katz, Ulf von Euler, and Julius Axelrod were awarded The Nobel Prize in Physiology or Medicine “for their discoveries concerning the humoral transmitters in the nerve terminals and the mechanism for their storage, release, and inactivation.”
*1971*	J. R. Vane; G. J. Roth et al., unveiled the mechanism of action of Aspirin.
*1971*	R. Miledi et al., isolated cholinergic receptor protein of torpedo electric tissue.
*1971*	Rodbell et al., showed the obligatory role of guanylnucleotides in Glucagon’s action.
*1971*	Earl W. Sutherland was awarded The Nobel Prize in Physiology or Medicine “for his discoveries concerning the mechanisms of the action of hormones.”
*1972*	J. F. R. Kerr et al., coined the term ‘Apoptosis.’
*1974*	Russel Ross et al., discovered that factors extracted from platelets could induce quiescent smooth muscle cells to synthesize DNA.
*1974*	A. Tissieres et al., demonstrated that temperature stress induces expression of Heat Shock Proteins.
*1975*	P. A. Lawrence and P. M. Shelton reported polarity in the developing insect retina.
*1975*	R. H. Michell showed that receptor-activated hydrolysis of PIP_2_ produced a molecule that caused an increase in intracellular calcium mobilization.
*1976*	J. F. Borel et al., showed immunosuppressive properties of macrolide Cyclosporine-A.
*1977*	Roger Guillemin and Andrew V. Schally were awarded The Nobel Prize in Physiology or Medicine “for their discoveries concerning the peptide hormone production of the brain.” Along with Rosalyn Yalow “for the development of radioimmunoassays of peptide hormones.”
*1977*	Y. Takai et al., confirmed the presence of Cyclic-nucleotide independent protein kinase in bovine cerebellum.
*1979*	Discovery of p53 signaling.
*1979*	H. N. Antoniades et al., purified Platelet Derived Growth Factor (PDGF).
*1979*	R. A Weinberg et al., showed that DNA isolated from chemically transformed rodent fibroblasts caused the morphologic transformation of mouse fibroblasts.
*1979*	Philip Cohen published seminal studies on protein phosphorylation, Phosphorylase kinase and role of phosphatases PPPLCA and GSK3β in the regulation of glycogen metabolism.
*1980*	Erikson et al.; Hunter and Sefton, isolated Protein tyrosine kinase v-Src.
*1980*	A. Hershko and A. Varshavsky’s laboratories independently elucidated detailed signaling associated with ubiquitin system; degradation signals (degrons) in short-lived proteins; and in vivo controls of protein fluxes.
*1980*	P. J. Novick et al., elucidated intracellular transport pathways in Yeast.
*1980*	Wieschaus and Nusslein-Volhard identified embryonic lethal, loss-of-function alleles of *Wingless* (*Wg*).
*1981*	J. E. Smart et al., discovered viral oncogene Src constituted a mutated tyrosine protein kinase.
*1981*	A. Roberts and M. Sporn discovered TGF-β.
*1981*	R. H. Michell et al., showed IP_3_ acts as a secondary messenger capable of traversing through the cytoplasm to the ER, thereby stimulating the release of Ca^2+^ into the cytoplasm.
*1981*	David H. Hubel and Torsten N. Wiesel were awarded The Nobel Prize in Physiology or Medicine “for their discoveries concerning information processing in the visual system.”
*1982*	J. A. Cooper et al., reported dimerization of PDGFR.
*1982*	P. Walter and G. Blobel identified Signal Recognition Particle (SRP) and later contributed to the understanding of Unfolded Protein Response (UPR).
*1982*	Sune K. Bergström, Bengt I. Samuelsson and John R. Vane were awarded The Nobel Prize in Physiology or Medicine 1982 “for their discoveries concerning prostaglandins and related biologically active substances.”
*1982*	Y. Barde et al., purified Brain-Derived Neurotrophic Factor (BDNF).
*1983*	K. Shimizu et al., discovered neuroblastoma cell line transforming gene product N-Ras.
*1983*	Sir J. Black and P. Leff used β blockers for the treatment of Angina pectoris.
*1984*	R. A. Cerione et al., demonstrated functional coupling between guinea pig-derived β-2-Adrenergic receptor and human guanine nucleotide-binding regulatory protein (NS) of adenylyl cyclase was sufficient to recapitulate a neurotransmitter responsive system.
*1985*	A. N. Hollenberg et al., 1985; S. Green et al., 1986 discovered nuclear superfamily of receptors: Human glucocorticoid and Oestrogen receptor.
*1985*	M. S. Brown and J. L. Goldstein were awarded The Nobel Prize in Chemistry “for their discoveries concerning the regulation of cholesterol metabolism.”
*1985*	G. Grynkiewicz et al., discovered Ca^2+^ indicators with greatly improved fluorescence properties.
*1985*	Richard O Hynes and J. W. Tamkun co-discovered Integrins.
*1985*	Lewis C Cantley and co-workers discovered Phosphoinositide kinase (PI3K).
*1986*	P. Russell and P. Nurse discovered Dual specificity phosphatases.
*1986*	Stanley Cohen and Rita Levi-Montalcini were awarded The Nobel Prize in Physiology or Medicine “for their discoveries of growth factors.”
*1986*	R. Weinberg elaborated the role of Ras, the first human cancer-causing gene and growth suppressor Retinoblastoma (Rb) protein.
*1986*	Ranjan Sen and David Baltimore discovered NF-ĸB.
*1986*	Tony Pawson and colleagues discovered the SH2 domain and its role in cellular transformation.
*1986*	U. Wilden and co-workers discovered arrestin.
*1986*	Y. Nishizuka et al., revealed that calcium released by IP_3_ work with DAG to activate protein kinase C.
*1987*	A. G. Gilman found that GTP cofactor acted through binding of “Transducer”, the effector protein that connects receptor and effector.
*1987*	S. J. Elledge and R. W. Davis discovered that ribonucleotide reductase (RNRs) are turned on by DNA damage and are regulated by the cell cycle.
*1987*	S. P. Staal cloned Akt oncogene and its human homologs AKT1 and AKT2.
*1987*	T. Imagawa et al., purified Ryanodine receptor from skeletal muscle sarcoplasmic reticulum.
*1987*	W. Lee et al., discovered AP-1 as a TPA-activated transcription factor that drives the expression of Metallothionein genes.
*1988*	R. A. Dixon et al., cloned first mammalian - β adrenergic receptor.
*1988*	Sir James W. Black, Gertrude B. Elion, and George H. Hitchings were awarded The Nobel Prize in Physiology or Medicine “for their discoveries of important principles for drug treatment.”
*1988*	K. J. Kemphues et al., discovered PAR proteins in *C. elegans* responsible for animal cell polarization.
*1988*	S. R. Sprang elucidated the mechanism of Glycogen Phosphorylase and kinase activation.
*1989*	E. Pfeuffer et al., discovered Olfactory Adenylyl Cyclase.
*1989*	S. G. Rhee et al., found that phospholipase C (PLC) is the phosphodiesterase responsible for hydrolyzing PIP_2_ into DAG and IP_3_.
*1989*	B. Vogelstein and co-workers discovered *TP53* to be the most frequently mutated in most human cancers.
*1989*	C. A. Finlay et al., showed that p53 proto-oncogene acts as a suppressor of transformation.
*1989*	Napoleone Ferrara discovered Vascular Endothelial Growth Factor (VEGF).
*1989*	P. Gardner showed the role of store-operated Ca^2+^ entry (SOCE), through STIM1 and ORAI1 in the understanding of immune cell activation (Clonal activation, and Tolerance).
*1989*	Sidney Altman and Thomas R. Cech were awarded The Nobel Prize in Chemistry 1989 “for their discovery of catalytic properties of RNA.”
*1990*	S. D. Wright et al., found that LPS sensing occurs through TLR4 and its coreceptor CD14.
*1991*	A. Galione et al., 1991; P. C. Lee et al., 1993 found that adenine nucleotides induced Ca^2+^ mobilization in the sea urchin.
*1991*	Erwin Neher and Bert Sakmann were awarded The Nobel Prize in Physiology or Medicine “for their discoveries concerning the function of single ion channels in cells.”
*1991*	B. Vogelstein and K. W. Kinzler discovered another tumor suppressor gene associated with familial adenomatous polyposis (FAP) and known as Adenomatous polyposis coli.
*1991*	C. I. Bargmann and H. R. Horvitz identified chemosensory neurons that are important in the olfactory sense in nematode *C. elegans*.
*1991*	F. Mckeon discovered immunosuppressant Tacrolimus/FK506 was a potent inhibitor of FKBP1A and inhibits calcineurin signaling by hindering substrate access.
*1991*	Hans Cleaver and co-workers reported the cloning of a T cell-specific transcription factor that they termed TCF1 and elaborated Wnt signaling.
*1991*	M. N. Hall and co-workers discovered the target of rapamycin (TOR) and its role in cell growth control in *Saccharomyces cerevisiae*.
*1991*	Richard R. Ernst was awarded The Nobel Prize in Chemistry “for his contributions to the development of the methodology of high resolution nuclear magnetic resonance (NMR) spectroscopy.”
*1992*	Edmond H. Fischer and Edwin G. Krebs were awarded The Nobel Prize in Physiology or Medicine “for their discoveries concerning reversible protein phosphorylation as a biological regulatory mechanism.”
*1992*	S. Nakanishi et al., 1992; G. Powis et al., 1994, identified A microbial product Wortmannin as an inhibitor of myosin light chain kinase and later as an inhibitor of PI3K.
*1992*	Yoshinori Ohsumi and coworkers discovered that autophagy also occurs in Yeast.
*1993*	J. P. Oliver et al., found that *Drosophila* SH2-SH3 adaptor protein is involved in coupling the sevenless tyrosine kinase to an activator of Ras guanine nucleotide exchange, Sos.
*1993*	Kazutoshi Mori identified a cellular quality-control system unfolded proteins response (UPR) and identified IRE1 as a core component of the UPR in Yeast.
*1993*	N. Marchenko et al., demonstrated a direct role of p53 in mitochondrial apoptosis.
*1994*	Alfred G. Gilman and Martin Rodbell were awarded The Nobel Prize in Physiology or Medicine “for their discovery of G-proteins and the role of these proteins in signal transduction in cells.”
*1994*	M. Rothe et al., identified TRAF-2 as Signal transducer associated with the cytoplasmic domain of the 75kDa tumor necrosis factor receptor 2.
*1997*	Paul D. Boyer, John E. Walker, and Jens C. Skou were jointly awarded The Nobel Prize in Chemistry “for their elucidation of the enzymatic mechanism underlying the synthesis of adenosine triphosphate (ATP).”
*1997*	Three groups independently discovered the SOCS1 protein: T. A. Endo et al., as a JAK-binding protein (JAB) as a suppressor of IL-6 signaling, R. Starr et al., based on sequence homology with the STAT3-SH2 domain and T. Naka et al., STAT-induced STAT Inhibitor (SSI).
*1998*	Robert F. Furchgott, Louis J. Ignarro and Ferid Murad were awarded The Nobel Prize in Physiology or Medicine “for their discoveries concerning nitric oxide as a signaling molecule in the cardiovascular system.”
*1999*	Günter Blobel was awarded The Nobel Prize in Physiology or Medicine “for the discovery that proteins have intrinsic signals that govern their transport and localization in the cell.”
*2000*	Arvid Carlsson, Paul Greengard, and Eric R. Kandel were awarded The Nobel Prize in Physiology or Medicine “for their discoveries concerning signal transduction in the nervous system.”
*2001*	Leland H. Hartwell, Tim Hunt, and Sir Paul M. Nurse were awarded The Nobel Prize in Physiology or Medicine “for their discoveries of key regulators of the cell cycle.”
*2002*	J. Takagi et al., investigated how integrin binding is conveyed to the cell interior.
*2002*	K. Hamada et al., 3D structures of the inositol 1,4,5-triphosphate (IP_3_) were elucidated.
*2002*	P. Lassus et al., the apoptosome may act as an amplifier rather than an initiator of caspase activation.
*2003*	Peter Agre and Roderick MacKinnon were jointly awarded The Nobel Prize in Chemistry “for the discovery of water channels” and “for structural and mechanistic studies of ion channels” respectively.
*2003*	B. D. Manning et al., Identification of tumor suppressor gene product tuberin (tuberous sclerosis complex-2) as a target of the phosphoinositide 3-kinase/Akt pathway.
*2003*	M. Yaffe and Coworkers identified BRCT repeats as phosphopeptide-binding modules involved in protein targeting.
*2004*	G. Di Paolo et al., provided genetic evidence for a critical role of PI(4,5)P2 synthesis in the physiology of neurotransmission.
*2004*	I. Tassiulas et al., Inflammatory responses in macrophages by Syk and ITAM-containing adaptors were reported.
*2004*	Richard Axel and Linda B. Buck were awarded The Nobel Prize in Physiology or Medicine “for their discoveries of odorant receptors and the organization of the olfactory system.”
*2004*	Aaron Ciechanover, Avram Hershko and Irwin Rose were awarded The Nobel Prize in Chemistry “for the discovery of ubiquitin-mediated protein degradation.”
*2005*	Edward S. Boyden accredited with discovering the Optogenetics.
*2005*	R. B. Seth et al., Identification and characterization of MAVS, a mitochondrial antiviral signaling protein that activates NF-κB and IRF-3.
*2005*	J. Liou et al., identified STIM as a Ca^2+^ sensor essential for Ca^2+^-store-depletion-triggered Ca^2+^ influx.
*2005*	J. Ptacek et al., Systems-level macromolecular networks in Yeast were identified by Globalanalysis of protein phosphorylation.
*2006*	Roger D. Kornberg was awarded The Nobel Prize in Chemistry “for his studies of the molecular basis of eukaryotic transcription.”
*2006*	S. Takamori et al., presented Molecular anatomy of a trafficking organelle.
*2007*	M. J. Rust et al., presented the role of ordered phosphorylation in the oscillation of a three-protein circadian clock in *Cyanobacteria*.
*2007*	S. Y. Zhang TLR3 deficiency was found to be associated with susceptibility to herpes simplex encephalitis.
*2007*	J. Bilic et al., LRP6-signalosome” is depended on scaffolding protein Dishevelled.
*2008*	D. Pincus et al., discussed the evolution of phospho-tyrosine signaling machinery in Premetazoan lineages.
*2008*	B. Apsel et al., accredited with the discovery of dual inhibitors of tyrosine and phosphoinositide kinases.
*2008*	Osamu Shimomura, Martin Chalfie and Roger Y. Tsien were awarded The Nobel Prize in Chemistry “for the discovery and development of the green fluorescent protein, GFP.”
*2009*	E. Meylan et al., reported connections of NF-κB and oncogenic K-Ras in lung tumor development.
*2009*	W. L. Yang et al., Membrane recruitment and phosphorylation of ubiquitinated AKT are promoted by TRAF6.
*2010*	A. Breitkreutz et al., deciphered A Global Protein Kinase and phosphatase interaction network in Yeast.
*2010*	S. J. Heidorn showed that kinase-dead BRAF cooperates with Ras to hyperactivated CRAF leading to enhancement of MEK and ERK signaling.
*2010*	J. Oh et al., showed an association of mTORC2 with ribosomes and phosphorylates the nascent Akt peptide.
*2011*	B. M. Gardner and P. Walter described that the unfolded proteins themselves bind directly to Ire1 to stimulate their oligomerization and activation.
*2011*	B. Gerlach et al., Linear ubiquitination prevents inflammation and regulates immune signaling.
*2011*	E. A. Kiss et al., Natural aryl hydrocarbon receptor ligands control organogenesis of intestinal lymphoid follicles.
*2012*	Eric Kandel and group discovered piRNAs as epigenetic controllers of memory-related synaptic plasticity.
*2012*	T. R. Wilson et al., highlighted the widespread potential for growth-factor-driven resistance to anticancer kinase inhibitors.
*2012*	Robert J. Lefkowitz and Brian K. Kobilka were awarded The Nobel Prize in Chemistry “for studies of G-protein-coupled receptors.”
*2012*	Sir John B. Gurdon and Shinya Yamanaka were awarded The Nobel Prize in Physiology or Medicine “for the discovery that mature cells can be reprogrammed to become pluripotent.”
*2013*	James E. Rothman, Randy W. Schekman and Thomas C. Südhof were awarded The Nobel Prize in Physiology or Medicine “for their discoveries of machinery regulating vesicle traffic, a major transport system in our cells.”
*2013*	James Chen and colleagues discovered another second messenger cGAMP and its activation through Cyclic GMP-AMP synthase, a cytosolic DNA sensor that activates the Type-I interferon pathway and confers antiviral immunity.
*2013*	J. E. Toettcher et al., employed optogenetics to interrogate the dynamic control of signaling transmission by the Ras/Erk module.
*2013*	P. Vizán et al., revealed Receptor dynamics determine attenuation and refractory behavior of the TGF-β pathway.
*2014*	J. C. H. Tam et al., highlighted Intracellular sensing of complement C3 activates cell-autonomous immunity.
*2014*	M. AlQuraishi et al., used a computational approach to establish a statistical mechanical framework that integrates biophysical and genomic data to assemble cancer networks.
*2015*	Tomas Lindahl, Paul Modrich and Aziz Sancar were awarded The Nobel Prize in Chemistry 2015 “for mechanistic studies of DNA repair.”
*2015*	R. G. Efremov deciphered architecture and conformational switch mechanism of the ryanodine receptor.
*2015*	M. Lazarou et al., highlighted the role of the ubiquitin kinase PINK1 in autophagy receptors to induce mitophagy.
*2016*	John Sondek designed a peptide based on Helix-Turn-Helix (HTH) that selectively blocks an important class of Gα_q_ proteins and prevents interaction with its downstream signaling partners.
*2016*	Nicolas Doucet and his research team found that activated RTKs terminate downstream signaling via the direct phosphorylation of an evolutionarily conserved Tyr present in most SRC homology (SH) 3 domains.
*2017*	Barry V. L. Potter and Andreas H. Guse and their co-workers found that 2′-deoxy-ADPR (dADPR) an endogenous TRPM2 superagonist may act as a cell-signaling molecule.
*2017*	R. Ravindran et al., discovered that the amino acid sensor GCN2 controls gut inflammation by inhibiting inflammasome activation.
*2017*	R. A. Saxton et al., showed the structural basis for leucine sensing by the Sestrin2-mTORC1 pathway.
*2018*	Frances H. Arnold and George P. Smith were awarded The Nobel Prize in Chemistry “for the directed evolution of enzymes” and “for the phage display of peptides and antibodies” respectively.
*2018*	Nicolas Doucet identified a discovery of a new molecular switch that controls activated receptor tyrosine kinases (RTKs) through SRC homology (SH) 3 domains.
*2018*	John Sondek and Team developed small proteins called peptides that selectively block a certain type of G-protein signaling.
*2019*	Jay T. Groves and his team showed that a molecular assembly phase transition and kinetic proofreading modulate Ras activation by Sos.
*2019*	Kaisa Lehti et al., found that FGFR4 efficiently phosphorylates several essential proteins of the Hippo tumor suppressor pathway.

Note—Due to space limitation, a few discoveries are left out [Sources for the table: https://www.nobelprize.org/; https://www.ncbi.nlm.nih.gov/pubmed; https://stke.sciencemag.org (Signaling Breakthroughs of the Year)].

**Table 3:** Major approaches and techniques used in cell signaling studies.

MAJOR APPROACHES	TECHNIQUES
*Imaging*	Light microscopy. Electron microscopy. Confocal microscopy to study fluorescently labelled proteins and indirect immunofluorescence. Wide-field Fluorescent microscopy. Total internal reflection fluorescence microscopy (TIRFM) for imaging highly dynamic clusters. Photoactivated localization microscopy (PALM). Stochastic optical reconstruction microscopy (STORM). Raster image correlation spectroscopy (RICS) employed for molecular diffusion and binding analysis. Correlative light and electron microscopy. Visualization of functions of intracellular organelle function using fluorescent probes.
*Centrifugation*	Density gradient centrifugation.
*Flow cytometry*	Multiplex flow cytometry.
*Chromatography*	Ion Exchange Chromatography. Affinity Chromatography. Gel Filtration Chromatography
*Spectroscopy*	Circular Dichroism. Optical rotatory dispersion. NMR Spectroscopy.
*Algorithms*	IQR algorithm. ConCavity. High-Performance Multi-Objective Evolutionary Algorithm (HPMOEA). Synthetic Genetic logic circuits.
*Transcriptomics and metabolomics characterization*	Single-cell RNA sequencing (scRNA-seq). Microarray analysis. NanoString Counter analysis. Ingenuity Systems Pathways Analysis (IPA).
*Protein-protein interactions* *Protein-nucleic acid interactions*	Yeast-two-hybrid.RNA sequencing. Western blotting and co-immunoprecipitation. In vitro phosphorylation assays. Phosphoproteomics. Mass spectrometry. 2-D gel electrophoresis. Microchannel for multiparameter analysis of proteins in a single complex” (mMAPS). Structure analysis –X-ray crystallography.
*In vitro and* in vivo *real-time analysis*	Live cell (time-lapse) imaging using fluorescent reporter. Protein covalently linked to the coding region of the signaling protein of interest. In vivo imaging for enzyme activation. Visualizing changes in intracellular levels calcium in vivo in different regions of the brain utilizing genetically encoded calcium indicator. FRET (Förster Resonance Energy Transfer (FRET). FRAP Fluorescence Recovery after Photobleaching (FRAP).
